# Heat and mass transfer on MHD squeezing flow of Jeffrey nanofluid in horizontal channel through permeable medium

**DOI:** 10.1371/journal.pone.0250402

**Published:** 2021-05-06

**Authors:** Nur Azlina Mat Noor, Sharidan Shafie, Mohd Ariff Admon

**Affiliations:** Faculty of Science, Department of Mathematical Sciences, Universiti Teknologi Malaysia, Johor Bahru, Johor, Malaysia; Central University of Karnataka, INDIA

## Abstract

The heat and mass transfer on time dependent hydrodynamic squeeze flow of Jeffrey nanofluid across two plates over permeable medium in the slip condition with heat generation/absorption, thermal radiation and chemical reaction are investigated. The impacts of Brownian motion and thermophoresis is examined in the Buongiorno’s nanofluid model. Conversion of the governing partial differential equations to the ordinary differential equations is conducted via similarity transformation. The dimensionless equations are solved by imposing numerical method of Keller-box. The outputs are compared with previous reported works in the journals for the validation of the present outputs and found in proper agreement. The behavior of velocity, temperature, and nanoparticles concentration profiles by varying the pertinent parameters are examined. Findings portray that the acceleration of the velocity profile and the wall shear stress is due to the squeezing of plates. Furthermore, the velocity, temperature and concentration profile decline with boost in Hartmann number and ratio of relaxation to retardation times. It is discovered that the rate of heat transfer and temperature profile increase when viscous dissipation, thermophoresis and heat source/sink rises. In contrast, the increment of thermal radiation reduces the temperature and enhances the heat transfer rate. Besides, the mass transfer rate decelerates for increasing Brownian motion in nanofluid, while it elevates when chemical reaction and thermophoresis increases.

## 1. Introduction

The wide applications of ultrahigh cooling devices in the industrial sectors is important to improve the thermal devices’ effectiveness. Therefore, the development of an advanced heat transfer fluid by dispersing the metallic nanoparticles in a conventional fluid was discovered by Choi and Eastman [1]. The new innovative fluid with high thermal efficiency is known as nanofluid. The addition of nanoparticles improves the thermal conductivity of conventional fluid; for instance, water, ethylene glycol or engine oil. Eastman *et al*. [2] conducted the experimental studies on the dispersion of copper nanoparticles in the ethylene glycol. They found that the thermal conductivity of ethylene glycol boosts up to 40% caused by the suspended nanoparticles enhance the capability of heat transfer in the flow. Nanofluid has been utilized in various applications such as vehicle cooling, reducing fuel in electric power plant and nanodrug delivery [3]. Wong and De Leon [4] stated that the usage of nanofluid coolants in the device leads to energy saving and emission reduction, which result in decreasing the production cost. Later, the relative velocity of nanoparticles and base fluid generated by seven slip mechanisms was analysed by Buongiorno [5]. He pointed out that only Brownian motion and thermophoresis are responsible in the increment of heat transfer process in nanofluid. Following the pioneer works of Buongiorno, many scientists started using Buongiorno’s model in the problem involving nanofluid flow [6–8].

Squeeze flow is induced by the compression of two plates with external applied stress. The idea of squeezing flow is implemented in various engineering applications, for example moving pistons, lubrication systems, injection moulding and hydraulic lifts. The flow of non-Newtonian fluid within two moving surfaces was first investigated by Stefan [9] using theory of lubrication. Subsequent to the Stefan’s work, the research on squeeze flow has gain interest from researchers and it is discovered in different geometries. Later, Reynolds [10] and Archibald [11] explored the squeeze flow in the elliptical and rectangular geometries. The governing equations is formulated according to the Reynolds equation. However, it is not proper to be applied in the squeezing flow with high velocity and porous thrust bearings as stated in Jackson [12] and Ishizawa [13] studies. Therefore, many renewed studies are performed to revise the formulation model of the squeeze flow mathematically [14–21].

Jeffrey fluid is classified as Non-Newtonian fluid due to the flow behaviour depends on the shear stress applied. The fluid acts as a solid if the shear stress exerted is lower than yield stress, whereas the fluid begins to flow if the shear stress exerted is more than yield stress [22,23]. The mathematical model of Jeffrey fluid used time derivatives as an alternative to convective derivatives. The parameter of relaxation and retardation times in the Jeffrey model are significant for the representation of the viscoelastics properties in the polymer sectors [24]. Jeffrey fluid model is treated as the model for the blood flow over thin arteries [25], motion of chyme through small intestine [26] and food bolus in oesophagus [27] due to its rheological characteristics.

The study of magnetohydrodynamics flow at the boundary layer has been acknowledged by many scientists due to its concept is applicable in various engineering devices. The magnetic field is imposed in the electrical conducted fluid generate Lorentz force. The applications of Lorentz force occur in various devices such as mass spectrometers and cyclotrons [28,29]. Recently, the advancement of Darcy’s Law lead to the research on the flow through porous medium increases. The implementation of porous medium is recommended in the engine cooling devices because it is useful in boosting the heat removal in the devices [30]. Hayat *et al*. [31] studied the impact of MHD on unsteady Jeffrey fluid flow caused by squeeze between two porous plates with suction and injection. The analytical series solution was achieved by Homotopy analysis method (HAM). Then, Muhammad *et al*. [32] extended Hayat *et al*. [31] with the stretching porous lower surface. The MHD flow of Jeffrey fluid through permeable medium across circular channel was analysed by Nallapu and Radhakrishnamacharya [33]. Later, the mixed convection flow of Jeffrey fluid over stretched vertical plate at a stagnation point with magnetic field was examined by Ahmad and Ishak [34]. Keller-box scheme is employed to discretize the problem numerically. Hayat *et al*. [35] explored MHD squeezing flow of Jeffrey nanofluid over two disks.

The role of viscous dissipation in the flow and heat transfer of high velocity or viscosity fluid is relevant in numerous manufacturing processes, such as strand casting, hot rolling and high extrusion of polymer materials. Sheikholeslami *et al*. [36] initiated the research on time dependent squeeze flow of nanofluid within two surfaces with viscous dissipation using Tiwari and Das model. The similar problem was reported from Pourmehran *et al*. [37] and Gorgani *et al*. [38] by implementing different methods. Acharya *et al*. [39] extended the previous problem with magnetic field impact. Later, Azimi and Riazi [40] discovered the work of Acharya *et al*. [39] via Buongiorno’s nanofluid model. The influences of viscous dissipation and velocity slip on the MHD squeeze flow of nanofluid was examined by Singh *et al*. [41]. In Non-Newtonian fluid, numerical solution of unsteady flow of Jeffrey nanofluid past a stretched surface with the impacts of Brownian motion, thermophoresis and viscous dissipation was presented by El-Zahar *et al*. [42]. Fourth-Order Finite Difference Continuation Method (FFDCM) is used in their study. The hydromagnetic flow of Jeffrey nanofluid over a stretching sheet with viscous dissipation and joule heating was studied by Shahzad *et al*. [43].

Thermal radiation is the electromagnetic waves that emitted from the heated surface. It is converted from thermal energy to electromagnetic energy due to the kinetic interactions among particles on the surface. The radiative heat transfer effects is usually arised in removal of heat on nuclear fuel debris, propulsion system and radiative waste materials disposal [44,45]. Madaki *et al*. [46] analysed the influences of thermal radiation and viscous dissipation on the squeeze flow of nanofluid within two plates by imposing Tiwari and Das model. Later, Sheikholeslami *et al*. [47] continued the work of Madaki *et al*. [46] by taking into account magnetic field. The previous problem was extended by Mittal *et al*. [48] with the impact of heat sink/source. Pandey and Kumar [49] investigated the unsteady MHD squeeze flow of nanofluid over porous medium with suction and injection. In Non-Newtonian fluid, Ashraf *et al*. [50] discovered the impacts of thermal radiation on MHD flow of Jeffrey nanofluid past a radial stretched wall with convective heat and mass transfer. The magnetohydrodynamic nanofluid flow on a stretching plate under the effects of thermal radiation, viscous dissipation and heat source/sink was reported by Sharma and Gupta [51].

The flow behaviour with chemical reaction is a topic of currrent interest because of its industrial importance, for instance nuclear power plants, chemical catalytic reactors, gas turbines and propulsion devices in aircraft [52,53]. Several researchers discovered the impacts of chemical reaction in the different flow geometries. The presence of chemical reaction and thermal radiation on hydromagnetic squeeze flow of nanofluid in two disks with suction and injection was discovered by Ullah *et al*. [54]. Further, Mohamed *et al*. [55] discussed the squeezing nanofluid flow across permeable medium with chemical reaction, thermal radiation, viscous dissipation and heat source/sink. In non-Newtonian fluid, the mixed convection flow of MHD Jeffrey nanofluid past permeable cone under the effects of chemical reaction and thermal radiation was examined by Raju *et al*. [56]. Later, Shankar and Naduvinamani [57] analysed the squeeze flow of Casson nanofluid with magnetic field, viscous dissipation, joule heating and chemical reaction. The work of Shankar and Naduvinamani [57] was continued by Noor *et al*. [58] with the flow of Casson nanofluid over porous medium in the presence of heat source/sink.

The abovementioned cited papers reveal that most of the studies limited to the squeeze flow of nanofluid. Clearly, no study is conducted on Jeffrey nanofluid flow between two moving plates. Motivated by the above literature survey, the objective of this research is to investigate unsteady squeeze flow of Jeffrey nanofluid with impacts of viscous dissipation, joule heating, chemical reaction, thermal radiation and heat source or sink. The numerical results are solved via Keller-box scheme and computed by MATLAB software. The graphical outputs for velocity, temperature and nanoparticles concentration are investigated with several important parameters.

The present work is applicable to be implemented as the model for nuclear reactor safety because it is involved the flow with chemical reaction [59]. The nuclear reaction process is automatically terminated when hazards occur. Furthermore, the presence of magnetic field in the flow increase the electricity induced by nuclear reactor [60]. Heat transfer capacity in nanofluid is higher than conventional fluid by thousand times. The addition of nanoparticles boosts the thermal conductivity of Jeffrey fluid. It is discovered that efficiency of heat transfer in nuclear power plant rises and consequently, decrease the thermal hydraulic problems system involving high temperature fluctuations due to thermal striping and thermal stratifications [61]. Hence, the new gateway for the better energy optimization and protection system in nuclear power plant is explored within the present study.

## 2. Mathematical formulation

**Fig 1:**
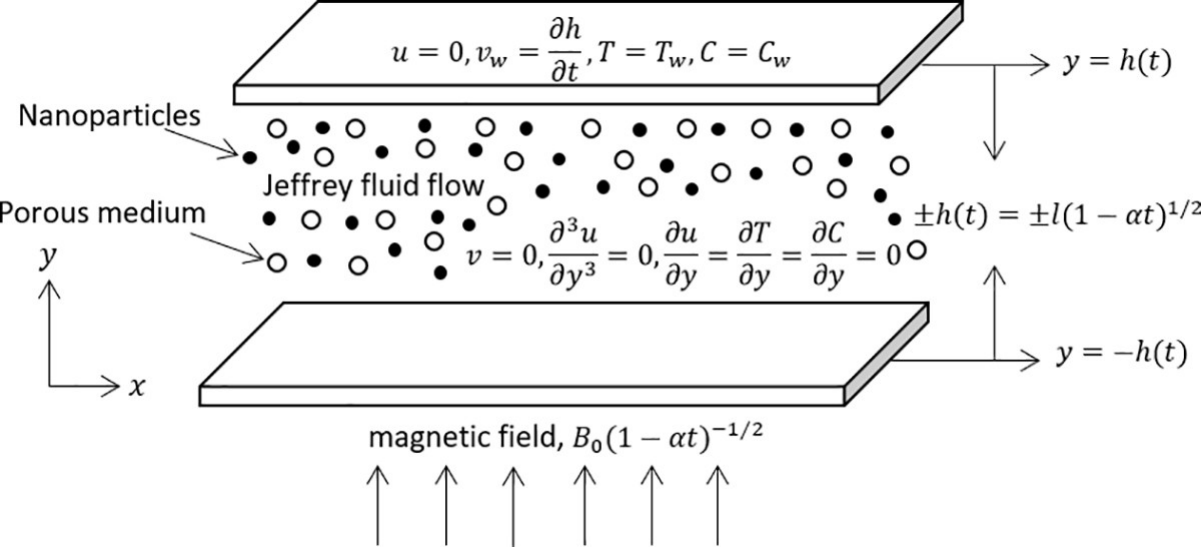
Coordinate system and geometrical model.

The unsteady magnetohydrodynamics flow of Jeffrey nanofluid due to squeeze of two surfaces over permeable medium with thermal radiation, chemical reaction and heat source/sink. Furthermore, the impacts of viscous dissipation and joule heating are analysed. The distance betweeen both plates is $y=\pm h(t)=\pm l{\left(1-\alpha t\right)}^{\frac{1}{2}}$. The upper and lower approaching closer with velocity $v_{w}(t)=\frac{\partial h(t)}{\partial t}$. The two plates are separated when *α*<0 and the plates are compressed when *α*>0 until *t* = 1/*α* [62,63]. The lower surface is imposed with magnetic field *B*(*t*) = *B*_0_(1−*αt*)^−1/2^ perpendicularly. Fig 1 presents the coordinate system and geometrical model for the squeeze flow of Jeffrey nanofluid.

**Table:** 

Nomenclature			
*B*	magnetic field	*T*_*w*_	temperature at upper plate (*K*)
*C*	nanoparticles concentration	*T*_*m*_	ambient temperature (*K*)
*C*_*w*_	concentration at upper plate	*t*	Time (*s*)
*c*_*f*_	specific heat of the fluid (*Jkg*^−1^ *K*^−1^)	*u*	flow velocity in *x* direction (*ms*^−1^)
*c*_*p*_	specific heat of nanoparticles (*Jkg*^−1^ *K*^−1^)	*v*	flow velocity in *y* direction (*ms*^−1^)
*D*_*B*_	Brownian diffusion coefficient (*m*^2^/*s*)	*v*_*w*_	velocity at upper plate (*ms*^−1^)
*D*_*T*_	Thermophoretic diffusion coefficient (*m*^2^/*s*)	(*x*,*y*)	cartesian coordinates
*De*	Deborah Number		
*Da*	Darcy Number	*Greek symbols*	
*Ec*	Eckert Number	*α*	constant
*Ha*	Hartmann number	*α*_*f*_	thermal diffusivity of Jeffrey fluid (*Wm*^2^*J*^−1^)
*h*	distance between two plates (*m*)	*f*	dimensionless velocity
*k*_1_	permeability of porous medium	*θ*	dimensionless temperature
*k*_1*_	mean absorption coefficient (*m*^−1^)	*δ*	dimensionless length
*k*_*f*_	thermal conductivity of fluid (*Wm*^−1^*K*^−1^)	*η*	boundary layer thickness
*k*_*c*_	rate of chemical reaction	*γ*	heat generation/absorption
*Le*	Lewis number	*φ*	porosity of permeable medium
*l*	initial distance of two plates (*m*)	*ν*_*f*_	kinematic viscosity (*m*^2^*s*^−1^)
*N*_*b*_	Brownian motion parameter	*ρ*_*f*_	fluid density (*kgm*^−3^)
*N*_*t*_	thermophoresis parameter	*ρ*_*p*_	density of nanoparticles
*Pr*	Prandtl number	*σ*	electrical conductivity (*A*^2^*s*^3^*kg*^−1^*m*^−3^)
*Q*	heat generation or absorption coefficient	σ*	Stefan-Boltzmann constant (*Wm*^−2^*K*^−4^)
*q*_*r*_	radiative heat flux	*λ*_1_	ratio of relaxation and retardation times
*R*	chemical reaction parameter	*λ*_2_	retardation time
*R*_*d*_	thermal radiation	*τ*	ratio of heat capacities of nanoparticles and fluid
*S*	squeeze number	*ϕ*	dimensionless concentration
*T*	fluid temperature (*K*)		

Based on the boundary layer approximations, the continuity, momentum, energy and concentration equations of Jeffrey nanofluid are

$$\frac{\partial u}{\partial x}+\frac{\partial v}{\partial y}=0,$$ (1)

$$\frac{\partial u}{\partial t}+u\frac{\partial u}{\partial x}+v\frac{\partial u}{\partial y}=\nu_{f}\left(1+\frac{1}{\lambda_{1}}\right)\frac{\partial^{2}u}{\partial y^{2}}+\nu_{f}\frac{\lambda_{2}}{1+\lambda_{1}}\left(\begin{array}{c} \frac{\partial^{3}u}{\partial t\partial y^{2}}+u\frac{\partial^{3}u}{\partial x\partial y^{2}}\\ +v\frac{\partial^{3}u}{\partial y^{3}}-\frac{\partial u}{\partial x}\frac{\partial^{2}u}{\partial y^{2}}+\frac{\partial u}{\partial y}\frac{\partial^{2}u}{\partial x\partial y} \end{array}\right)-\frac{\sigma B^{2}\left(t\right)}{\rho_{f}}u-\nu_{f}\left(1+\frac{1}{\lambda_{1}}\right)\frac{\varphi }{k_{1}\left(t\right)}u,$$ (2)

$$\frac{\partial T}{\partial t}+u\frac{\partial T}{\partial x}+v\frac{\partial T}{\partial y}=\alpha_{f}\frac{\partial^{2}T}{\partial y^{2}}+\tau \left[D_{B}\frac{\partial C}{\partial y}\frac{\partial T}{\partial y}+\frac{D_{T}}{T_{m}}{\left(\frac{\partial T}{\partial y}\right)}^{2}\right]+\frac{\nu_{f}}{c_{f}}\left(1+\frac{1}{\lambda_{1}}\right)\left[4{\left(\frac{\partial u}{\partial x}\right)}^{2}+{\left(\frac{\partial u}{\partial y}\right)}^{2}\right]+\frac{\sigma B^{2}\left(t\right)}{{\left(\rho c\right)}_{f}}u^{2}-\frac{1}{{\left(\rho c\right)}_{f}}\frac{\partial q_{r}}{\partial y}+\frac{Q\left(t\right)}{{\left(\rho c\right)}_{f}}T,$$ (3)

$$\frac{\partial C}{\partial t}+u\frac{\partial C}{\partial x}+v\frac{\partial C}{\partial y}=D_{B}\frac{\partial^{2}C}{\partial y^{2}}+\frac{D_{T}}{T_{m}}\frac{\partial^{2}T}{\partial y^{2}}-k_{c}\left(t\right)C,$$ (4)

The correlated boundary conditions are

$$u=0,v=v_{w}=\frac{\partial h(t)}{\partial t},T=T_{w},C=C_{w},\;\mathrm{at}\;y=h(t),$$ (5)

$$\frac{\partial u}{\partial y}=0,\frac{\partial^{3}u}{\partial y^{3}}=0,v=0,\frac{\partial T}{\partial y}=0,\frac{\partial C}{\partial y}=0,\;\mathrm{at}\;y=0.$$ (6)

The definition of radiative heat flux, *q*_*r*_ based on Roseland approximation is stated by

$$q_{r}=\frac{-4\sigma^{*}}{3k_{1}^{*}}\frac{\partial T^{4}}{\partial y},$$ (7)

The linear function of temperature is described by term *T*^4^ because of the small temperature gradient in the fluid. Then, *T*^4^ is expanded in Taylor series about *T*_∞_ by neglecting the higher order terms gives

$$T^{4}≅4T_{\infty }^{3}T-3T_{\infty }^{4}.$$ (8)

The energy equation is derived using Eqs (7) and (8) as follows

$$\frac{\partial T}{\partial t}+u\frac{\partial T}{\partial x}+v\frac{\partial T}{\partial y}=\alpha_{f}\left(1+\frac{16\sigma^{*}T_{\infty }^{3}}{3k_{f}k_{1}^{*}}\right)\frac{\partial^{2}T}{\partial y^{2}}\tau \left[D_{B}\frac{\partial C}{\partial y}\frac{\partial T}{\partial y}+\frac{D_{T}}{T_{m}}{\left(\frac{\partial T}{\partial y}\right)}^{2}\right]+\frac{\nu_{f}}{c_{f}}\left(1+\frac{1}{\lambda_{1}}\right)\left[{{4(\frac{\partial u}{\partial x})}^{2}+(\frac{\partial u}{\partial y})}^{2}\right]+\frac{\sigma B^{2}(t)}{{\left(\rho c\right)}_{f}}u^{2}+\frac{Q(t)}{{\left(\rho c\right)}_{f}}T.$$ (9)

The non-dimensional variables are implemented to reduce the partial differential equations to ordinary differential equations [22];

$$u=\frac{\alpha x}{2(1-\alpha t)}f^{\prime }(\eta ),\;v=-\frac{\alpha l}{2\sqrt{\left(1-\alpha t\right)}}f(\eta ),\;\eta =\frac{y}{l\sqrt{\left(1-\alpha t\right)}},\;\theta =\frac{T}{T_{w}},\;\phi =\frac{C}{C_{w}},$$ (10)

Substitute variables (10) into Eqs (2), (4) and (9), the dimensionless forms are obtained

$$\left(1+\frac{1}{\lambda_{1}}\right)f^{iv}-S(\eta f^{\prime \prime \prime }+3f^{\prime \prime }+f^{\prime }f^{\prime \prime }-ff^{\prime \prime \prime })+\left(1+\frac{1}{\lambda_{1}}\right)\frac{De}{2}(\eta f^{v}+5f^{iv}+2f^{\prime \prime }f^{\prime \prime \prime }-f^{\prime }f^{iv}-ff^{v})-{Ha}^{2}f^{\prime \prime }-\left(1+\frac{1}{\lambda_{1}}\right)\frac{1}{Da}f^{\prime \prime }=0,$$ (11)

$$\frac{1}{Pr}\left(1+\frac{4}{3}R_{d}\right)\theta^{\prime \prime }+S(f\theta^{\prime }-\eta \theta^{\prime }+\gamma \theta )+Ec\left[(1+\frac{1}{\lambda_{1}})[{\left(f^{\prime \prime }\right)}^{2}+4\delta^{2}{\left(f^{\prime }\right)}^{2}]+{Ha}^{2}{\left(f^{\prime }\right)}^{2}\right]{+N}_{b}\phi^{\prime }\theta^{\prime }+N_{t}{\left(\theta^{\prime }\right)}^{2}=0,$$ (12)

$$\frac{1}{Le}\phi^{\prime \prime }+S(f\phi^{\prime }-{\eta \phi }^{\prime })+\frac{1}{Le}\frac{N_{t}}{N_{b}}\theta^{\prime \prime }-R\phi =0,$$ (13)

with the non-dimensional boundary conditions

$$f(\eta )=0,f^{\prime \prime }(\eta )=0,f^{iv}(\eta )=0,\theta^{\prime }(\eta )=0,\phi^{\prime }(\eta )=0,\;at\;\eta =0,$$ (14)

$$f(\eta )=1,f^{\prime }(\eta )=0,\theta (\eta )=1,\phi (\eta )=1\;at\;\eta =1.$$ (15)

The significant parameters in the non-dimensional equations are defined as

$$S=\frac{\alpha l^{2}}{2\nu_{f}},\;Ha=lB_{0}\sqrt{\frac{\sigma }{\rho_{f}\nu_{f}}},\;Da=\frac{k_{0}}{\varphi l^{2}},\;De=\frac{\alpha \lambda_{2}}{1-\alpha t},\;\delta =\frac{l}{x}{\left(1-\alpha t\right)}^{1/2},\;Pr=\frac{\nu_{f}}{\alpha_{f}},\;Ec=\frac{\alpha^{2}x^{2}}{4c_{f}T_{w}{\left(1-\alpha t\right)}^{2}},$$

$$R_{d}=\frac{4\sigma^{*}T_{\infty }^{3}}{k_{f}k_{1}^{*}},\;\gamma =\frac{2Q_{0}}{\alpha {\left(\rho c\right)}_{f}},\;Le=\frac{\nu_{f}}{D_{B}},\;N_{b}=\frac{\tau D_{B}C_{w}}{\nu_{f}},\;N_{t}=\frac{\tau D_{T}T_{w}}{\nu_{f}T_{m}},\;R=\frac{k_{2}l^{2}}{\nu_{f}}.$$

Physically, the movement of channel is portrayed by squeezing number with *S*>0 shows the plates approaches closer and *S*<0 shows the plates separates further. Deborah, Darcy and Hartmann numbers are important parameter for velocity profile. Furthermore, thermal radiation, Eckert number and heat generation/absorption parameters are used for regulation of temperature profile. The effect of chemical reaction is exhibited in the nanoparticles concentration profile. The flow in the simultaneous momentum and mass diffusion is described by Lewis number.

## 3. Results and discussion

The ordinary differential Eqs (8) to (10) with related boundary conditions (11) and (12) are derived via Keller-box scheme. The results are obtained and plotted graphically using algorithm built in MATLAB software. The proper prediction for the step size and boundary layer thickness is necessary to achieve the precise results. Here, Δ*η* = 0.01 and *η*_∞_ = 1 are considered. Difference of previous and present outputs of velocity, temperature, and concentration is called convergence criteria. The iteration process is stopped when it converges to 10^−5^ [64].

The computations for *S*, *λ*_1_, *Ha*, *Da*, *De*, *δ*, *Pr*, *Ec*, *R*_*d*_, *γ*, *Le*, *N*_*b*_, *N*_*t*_ and *R* are conducted to analyse the behaviour of velocity, temperature and nanoparticles concentration. Tables 1 to 3 depict the present outputs are compared with the previous outputs in the journal as limiting cases.

**Table 1:** Numerical outputs of −*f*′′(1) for *S* when *λ*_1_ →∞, *Da*→∞, *De* = *N*_*b*_ = 10^−10^, *Ha* = *γ* = *Ec* = *δ* = *R*_*d*_ = *R* = *N*_*t*_ = 0 and *Le* = *Pr* = 1.

−*f*′′(1)			
*S*	Wang [17]	Ahmed *et al*. [65]	Present outputs
−0.9780	2.235	2.1915	2.1917
−0.4977	2.6272	2.6193	2.6194
−0.09998	2.9279	2.9277	2.9277
0	3.000	3.000	3.000
0.09403	3.0665	3.0663	3.0664
0.4341	3.2969	3.2943	3.2943
1.1224	3.714	3.708	3.708

**Table 2:** Numerical outputs of −*f*′′(1), −*θ*′(1) and *ϕ*′(1) for *S* as *λ*_1_→∞, *Da*→∞, *γ* = *Ha* = *R*_*d*_ = *N*_*t*_ = 0, *δ* = 0.1, *De* = *N*_*b*_ = 10^−10^ and *Ec* = *Pr* = *Le* = *R* = 1.

*S*	Naduvinamani and Shankar [62]			Present outputs		
	−*f*′′(1)	−*θ*′(1)	−*ϕ*′(1)	−*f*′′(1)	−*θ*′(1)	−*ϕ*′(1)
2.0	4.167389	3.118551	0.701813	4.167412	3.118564	0.701819
0.5	3.336449	3.026324	0.744224	3.336504	3.026389	0.744229
0.01	3.007134	3.047092	0.761225	3.007208	3.047166	0.761229
-0.5	2.617404	3.129491	0.781402	2.617512	3.129556	0.781404
-1.0	2.170091	3.319899	0.804559	2.170255	3.319904	0.804558

**Table 3:** Numerical outputs of Nusselt number for *Pr* and *Ec* when *λ*_1_→∞, *Da*→∞, *N*_*b*_ = *De* = 10^−10^, *Ha* = *γ* = *R*_*d*_ = *N*_*t*_ = 0, *S* = 0.5, *δ* = 0.1 and *R* = *Le* = 1.

−*θ*′(1)					
*Pr*	*Ec*	Pandey and Kumar [49]	Mustafa *et al*. [63]	Sheikholeslami *et al*. [66]	Present outputs
0.5	1.0	1.522367	1.522368	1.522367	1.522401
1.0	1.0	3.026324	3.026324	3.026323	3.026389
2.0	1.0	5.980530	5.980530	5.980530	5.980652
5.0	1.0	14.43941	14.43941	14.43941	14.43965
1.0	0.5	1.513162	1.513162	1.513162	1.513194
1.0	1.2	3.631588	3.631588	3.631588	3.631667
1.0	2.0	6.052647	6.052647	6.052647	6.052778
1.0	5.0	15.13162	15.13162	15.13162	15.13194

The numerical outputs of skin friction coefficient for *S* are compared with Wang [17] and Ahmed *et al*. 65] in Table 1. The comparison of skin friction coefficient, Nusselt and Sherwood numbers for *S* are portrayed in Table 2 with Naduvinamani and Shankar [62]. Table 3 shows that the Nusselt number are compared with Pandey and Kumar [49], Mustafa *et al*. [63] and Sheikholeslami *et al*. [66] for *Pr* and *Ec* values. The good agreement is found from the present outputs in Tables 1–3.

**Fig 2:**
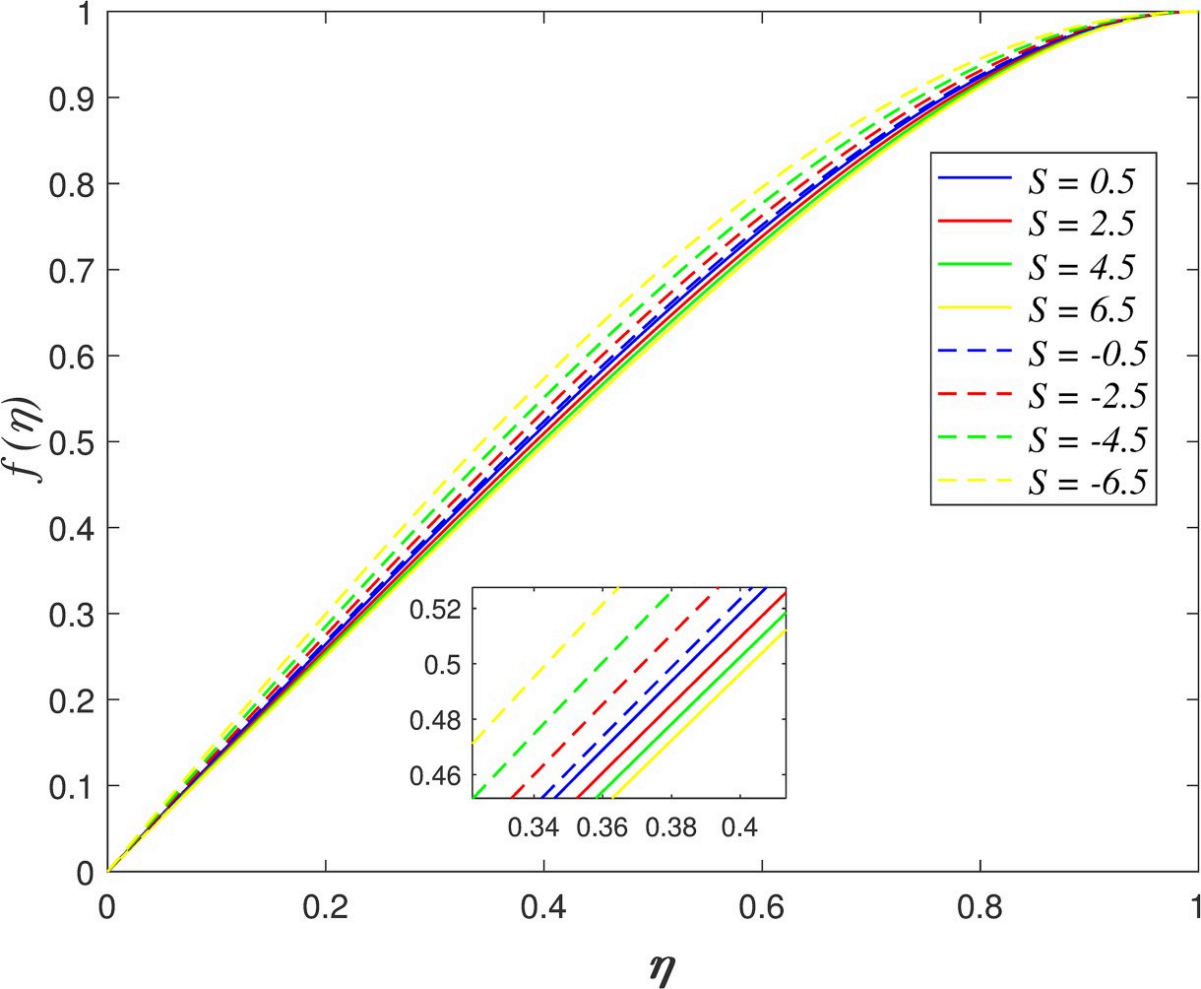
Impact of *S* on radial velocity.

**Fig 3:**
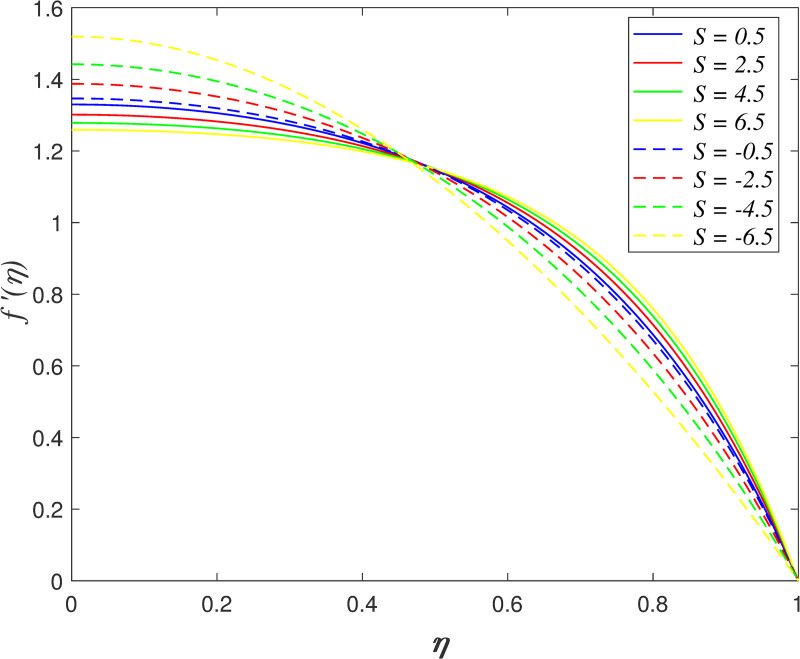
Impact of *S* on axial velocity.

**Fig 4:**
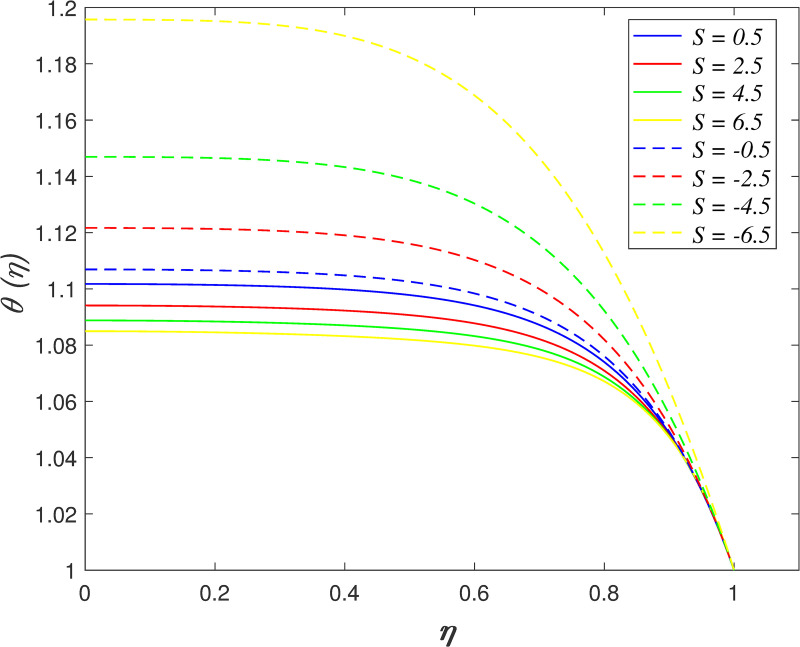
Impact of *S* on temperature.

**Fig 5:**
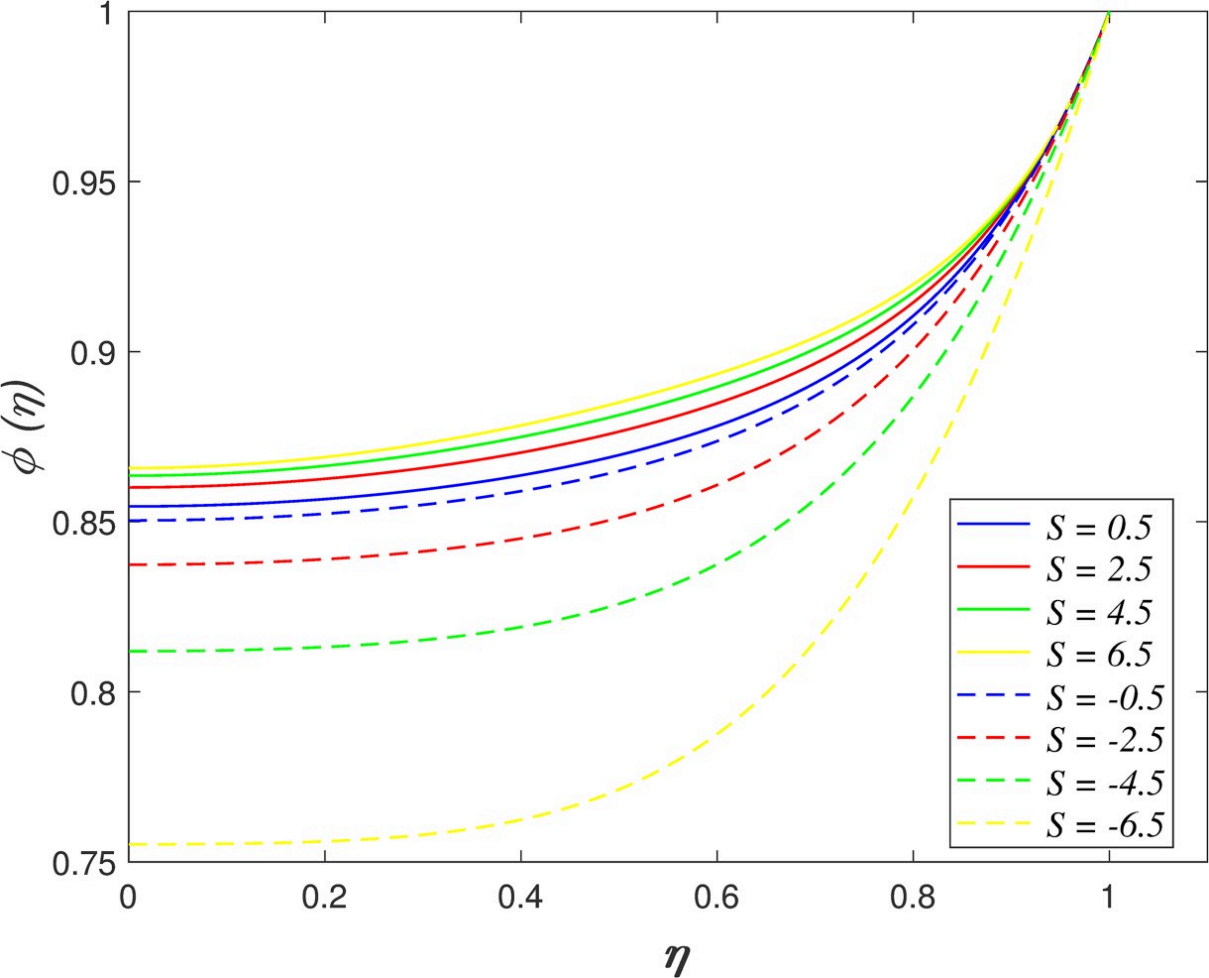
Impact of *S* on concentration.

Figs 2–5 portrays the influences of *S* on velocity, temperature, and concentration. The motion of plates towards one another is indicated by *S*>0 and the motion of plates further from one another is indicated by *S*<0. The radial velocity decelerating as *S*>0, and the velocity accelerating as *S*<0 as shown in Fig 2. Physically, the fluid is squeezed out from the channel as the surfaces moving nearer, which result in the velocity slowing down in the boundary area. On the contrary, the fluid is squeezed into the channel as the surfaces moving further, which result in increasing the velocity of the boundary area. Fig 3 portrays the variation of *S* on axial velocity. Generally, the area adjacent to the lower wall is 0≤*η*<0.45 and the area adjacent to the upper wall is 0.45≤*η*≤1. The velocity in the flow decreases for *η*<0.45 and it elevating for *η*≥0.45 when *S*>0. On the contrary, the velocity profile enhances for *η*<0.45 and it declines for *η*≥0.45 when *S*<0. It is discovered that the fluid across the narrow channel at a faster rate after the surfaces is compressed. Meanwhile, the velocity reducing because the fluid confronts additional resistance in the wider channel. The cross flow arises at the center of the boundary area. It is found that the velocity profile at the critical point *η*_*c*_ = 0.45 did not affected when varying the squeeze parameter. The impact of *S* on temperature field is demonstrated in Fig 4. It is shown that the temperature in the flow rises when *S*<0 due to the larger volume of the channel boost the kinetic energy of fluid particles. In contrast, the smaller volume of channel drops the kinetic energy of fluid particles and thus, decelerating the temperature field. The effect of *S* on nanoparticles concentration is illustrated in Fig 5. The concentration field rises when *S*>0 and it declines when *S*<0.

**Fig 6:**
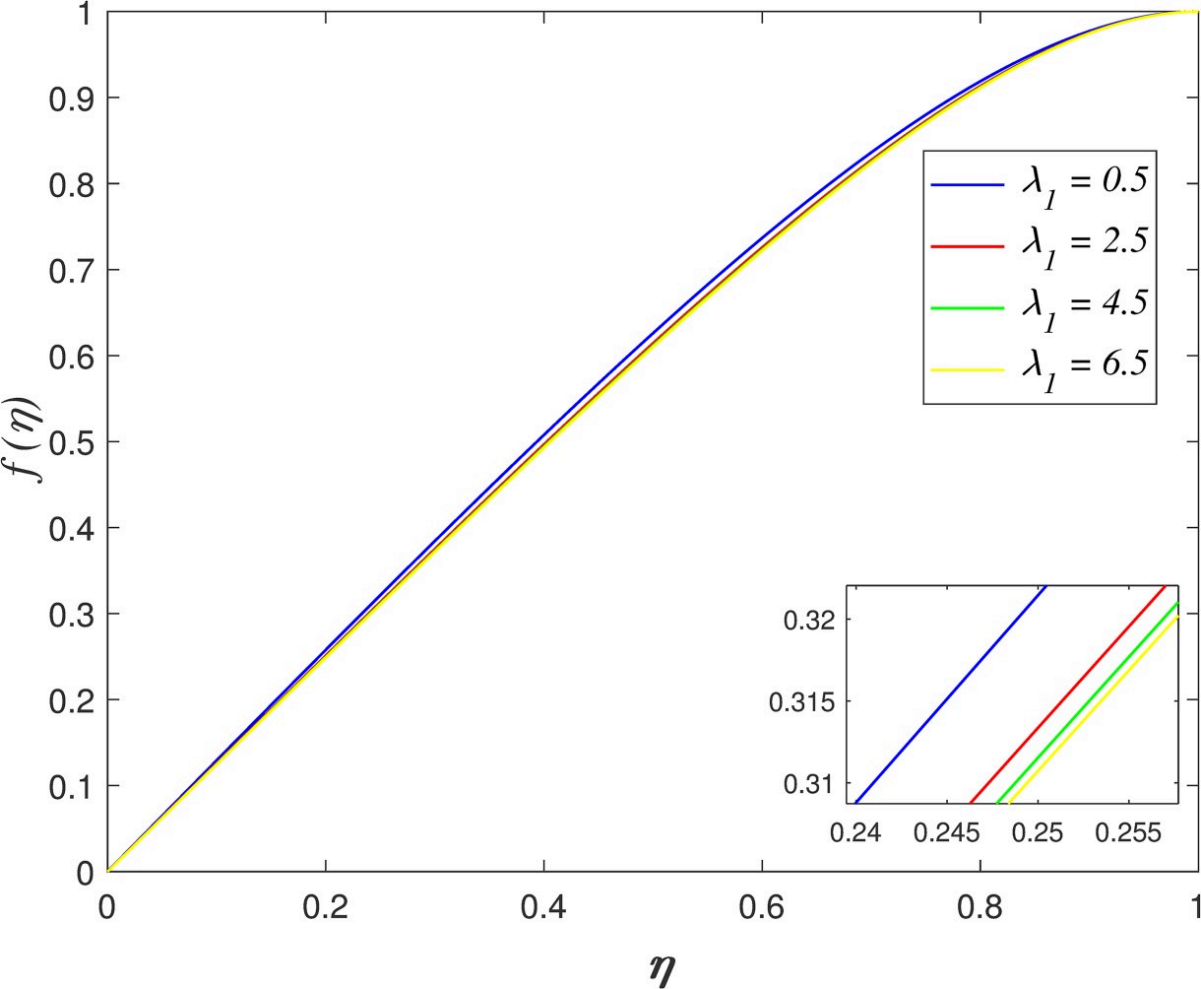
Impact of *λ*_1_ on radial velocity.

**Fig 7:**
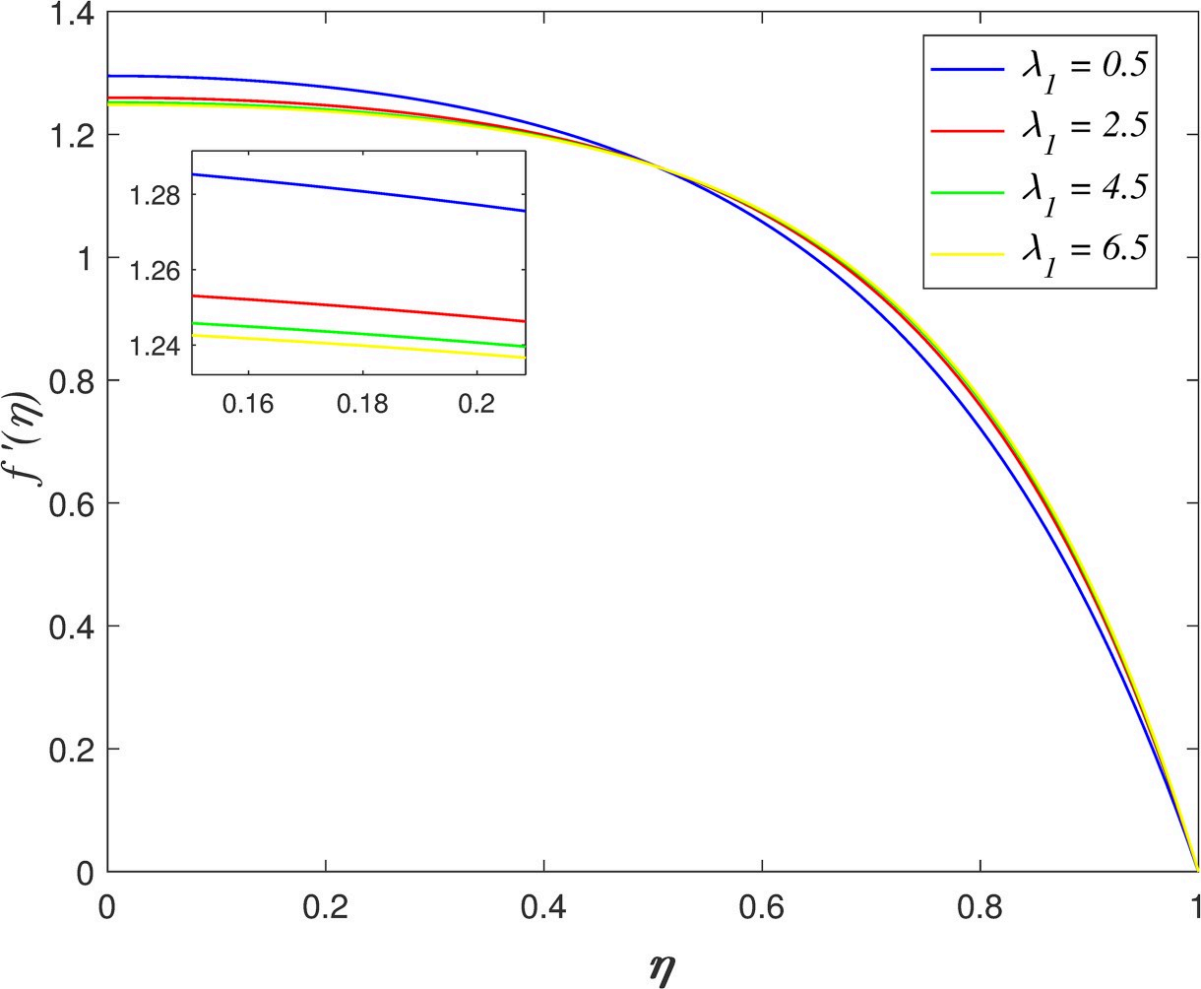
Impact of *λ*_1_ on axial velocity.

**Fig 8:**
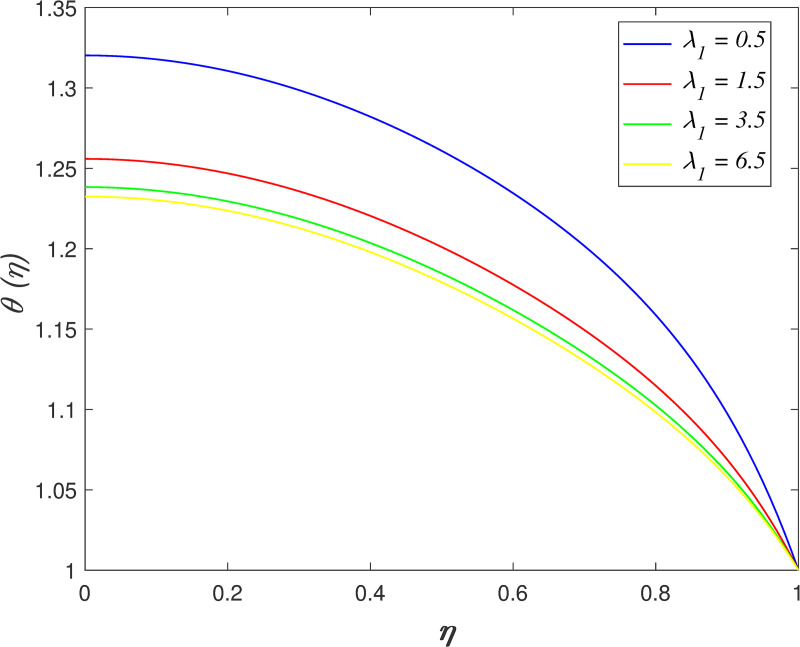
Impact of *λ*_1_ on temperature.

**Fig 9:**
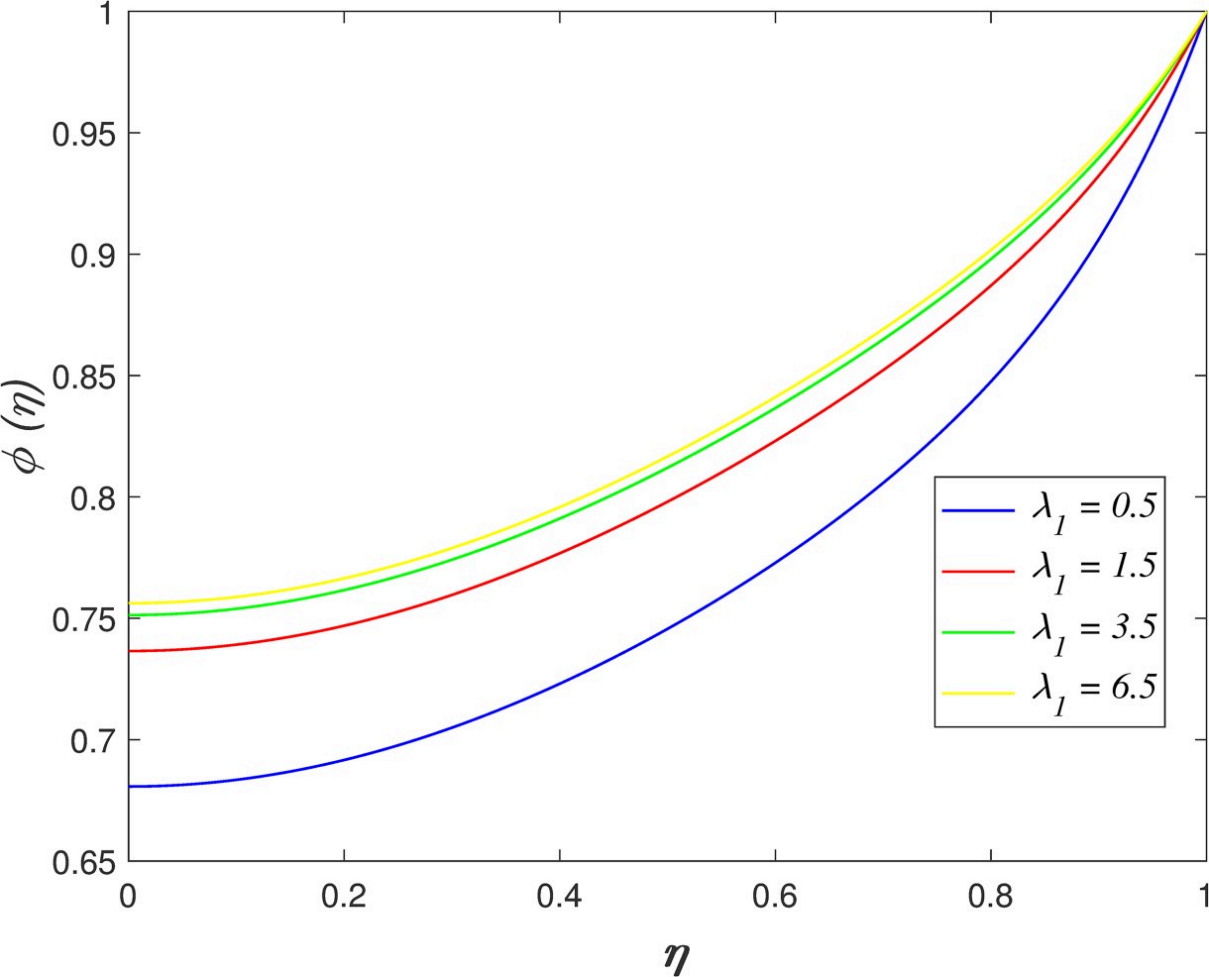
Impact of *λ*_1_ on concentration.

The impact of *λ*_1_ on velocity, temperature, and concentration is depicted in Figs 6–9. The radial velocity in Fig 6 decelerating for increasing *λ*_1_ values. The reason is that the increment of *λ*_1_ strengthen the intermolecular forces in the fluid particles and thus, increase the fluid viscosity within flow vicinity. Fig 7 portrays that the axial velocity slowing down for *η*≤0.5 and it increasing for *η*>0.5 as *λ*_1_ rises. The cross-behaviour of flow at the middle of the channel. Fig 8 presents the effect of *λ*_1_ on temperature field. It is noticed that flow temperature reduces with raise in *λ*_1_. The kinetic energy of fluid particles decelerating because of the high viscosity of fluid. The influence of *λ*_1_ on nanoparticles concentration is displayed in Fig 9. The enhancement of *λ*_1_ boosts the concentration profile.

**Fig 10:**
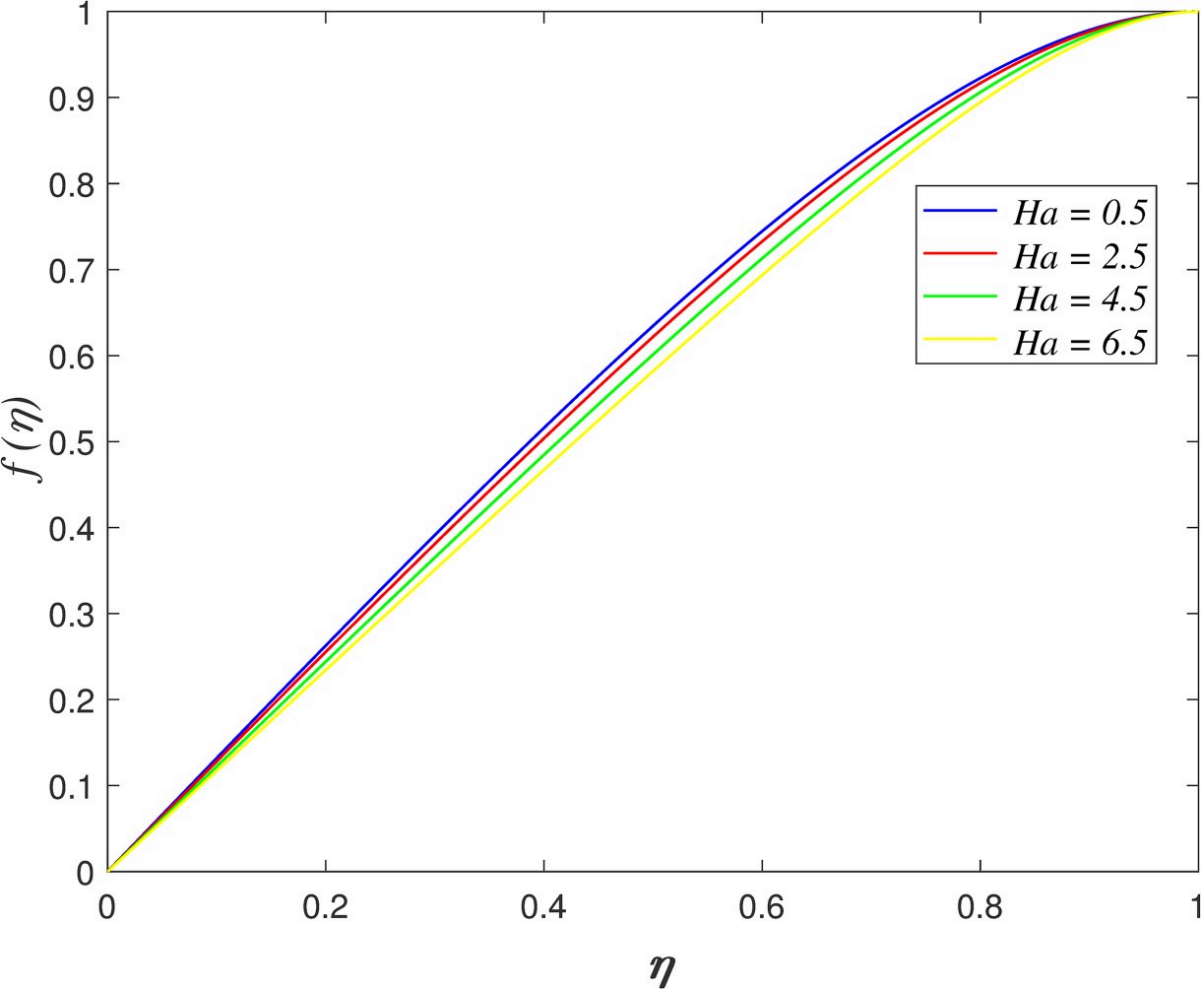
Impact of *Ha* on radial velocity.

**Fig 11:**
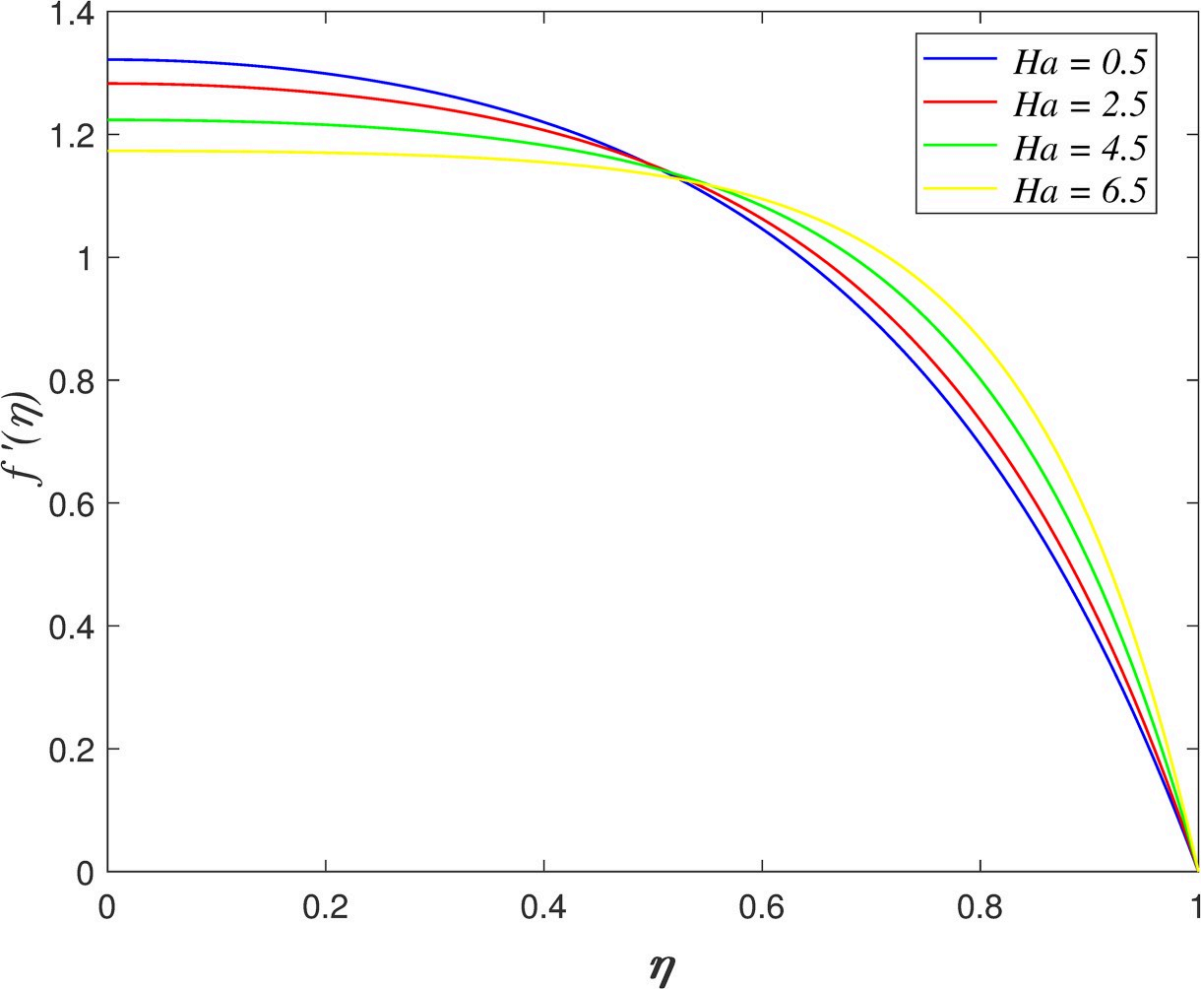
Impact of *Ha* on axial velocity.

**Fig 12:**
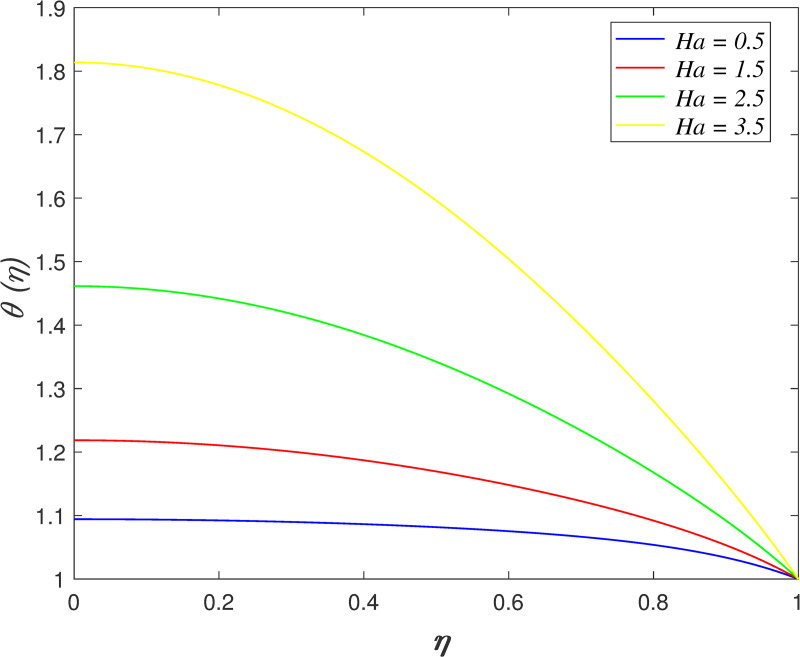
Impact of *Ha* on temperature.

**Fig 13:**
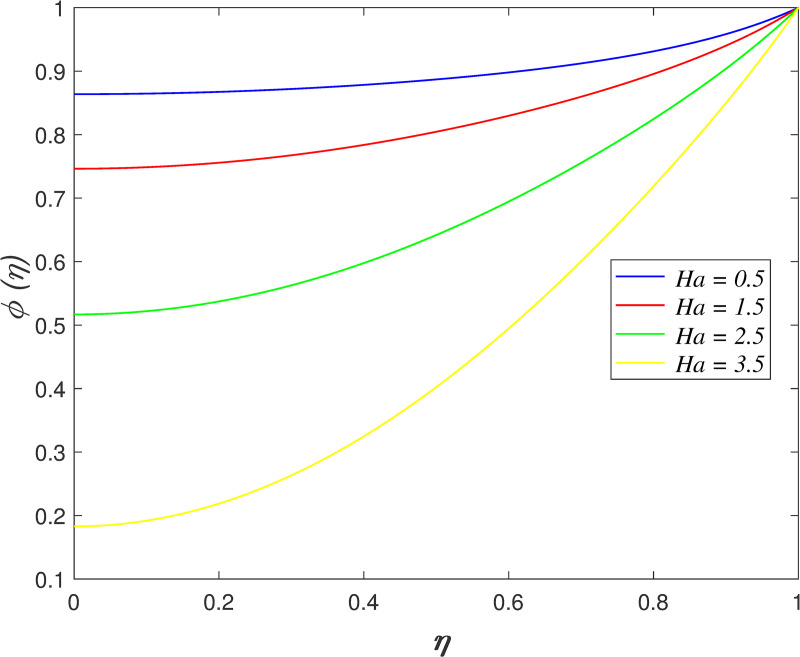
Impact of *Ha* on concentration.

The effect of *Ha* on velocity, temperature and concentration is illustrated from Figs 10–13. The flow velocity declines as shown in Fig 10 as *Ha* increases. It is discovered that the generation of Lorentz force caused by the magnetic field elevates the resistance in the flow area. Fig 11 displays that the axial velocity decreases for *η*≤0.5 and it enhancing for *η*>0.5 with raise in *Ha*. It is noticed that the cross flow of velocity profile occurs at the middle of the boundary layer. Fig 12 depicts the variation of *Ha* on temperature field. It is observed that the flow temperature boosts with raise in *Ha*. The influences of *Ha* on nanoparticles concentration is described in Fig 13. The reduction of concentration profile is shown for increasing *Ha*.

**Fig 14:**
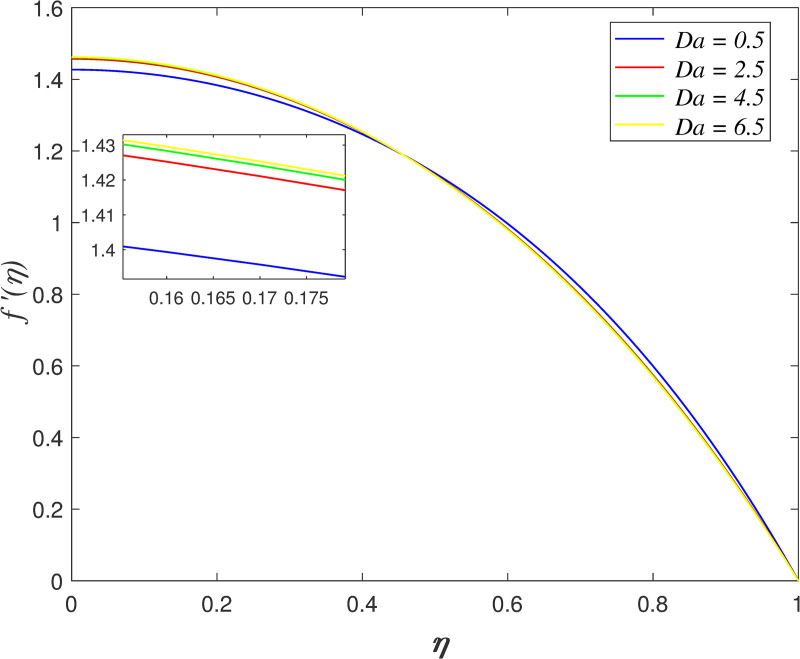
Impact of *Da* on axial velocity.

**Fig 15:**
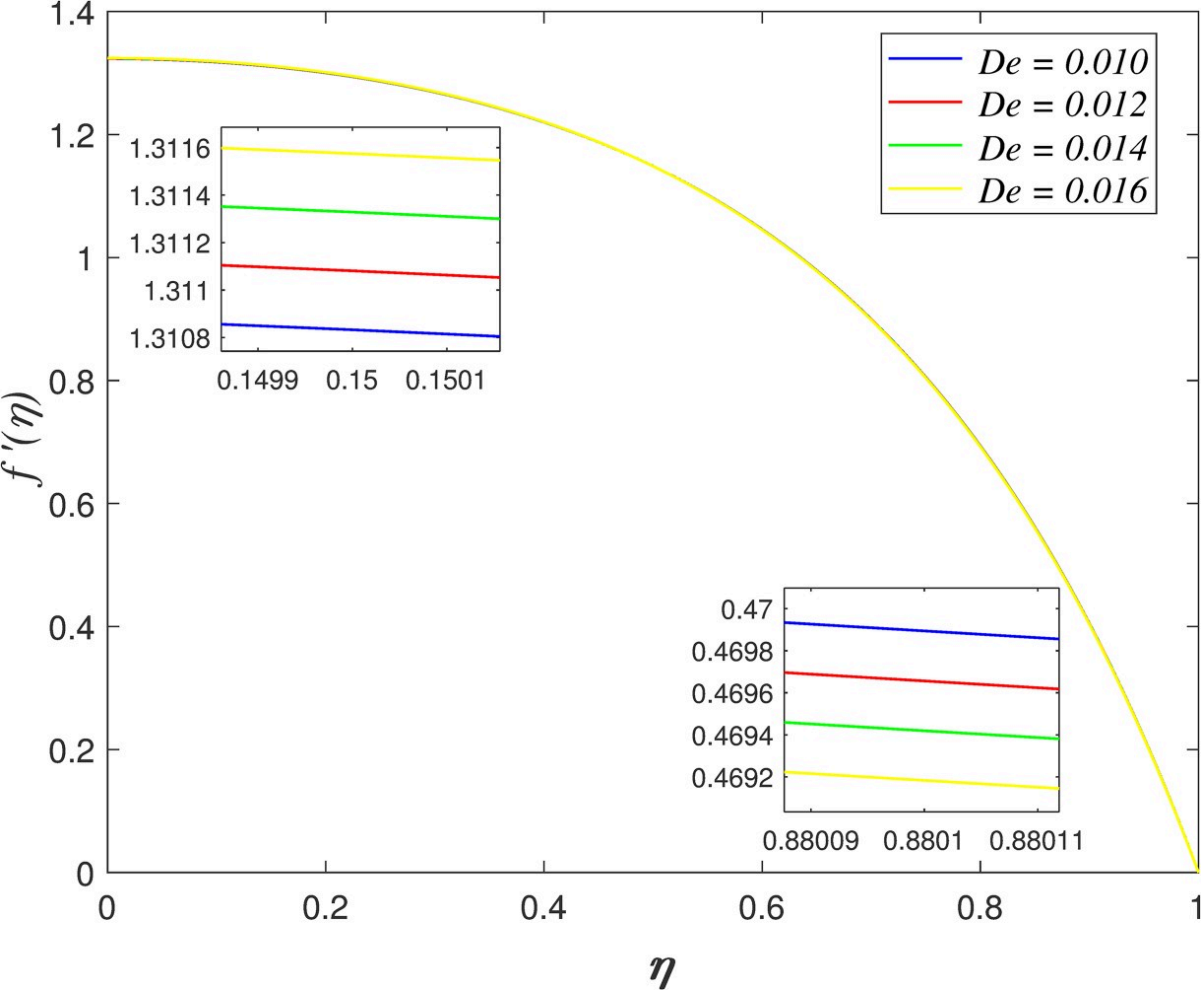
Impact of *De* on axial velocity.

Fig 14 presents the influence of *Da* on axial velocity. The velocity accelerating for *η*≤0.5 and it declining for *η*>0.5 as *Da* increases. The raise in Darcy number promotes the permeability of porous medium, which cause the flow across porous medium increasing. The effect of *De* on axial velocity is illustrated in Fig 15. It is discovered that the velocity increasing close to the lower wall and it decreases close to the upper wall with raise in *De*. The ratio of retardation time and observation time is described by Deborah number. Retardation time represents the slow reaction to the exerted stress or ‘delay of elasticity’. The raise of *De* implies that the flow has longer retardation time. This phenomenon boosts the viscosity of fluid and consequently, slowing down the flow adjacent to the upper wall.

**Fig 16:**
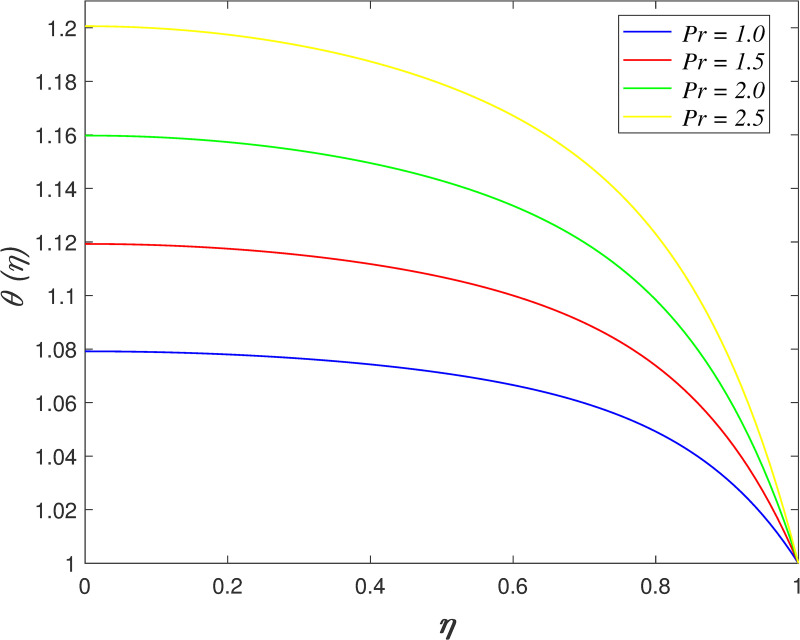
Impact of *Pr* on temperature.

**Fig 17:**
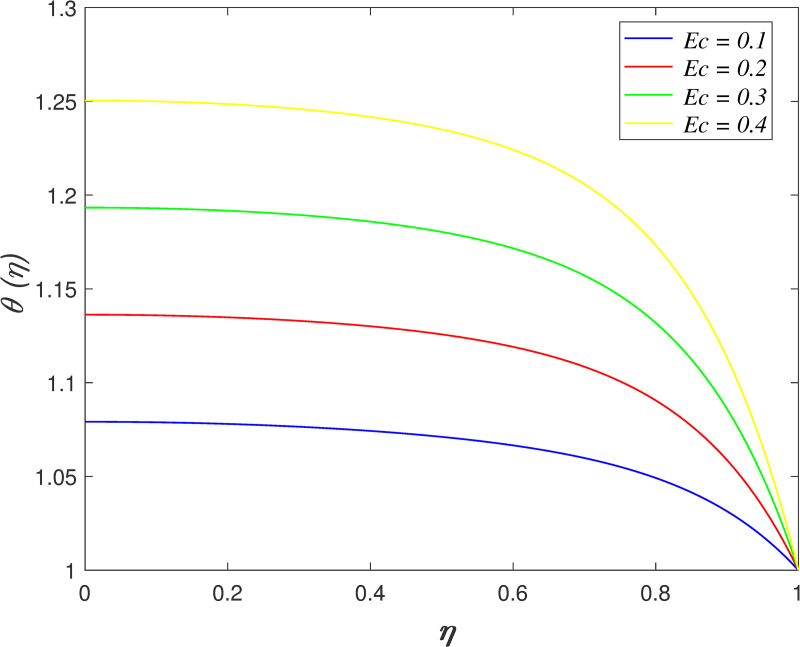
Impact of *Ec* on temperature.

**Fig 18:**
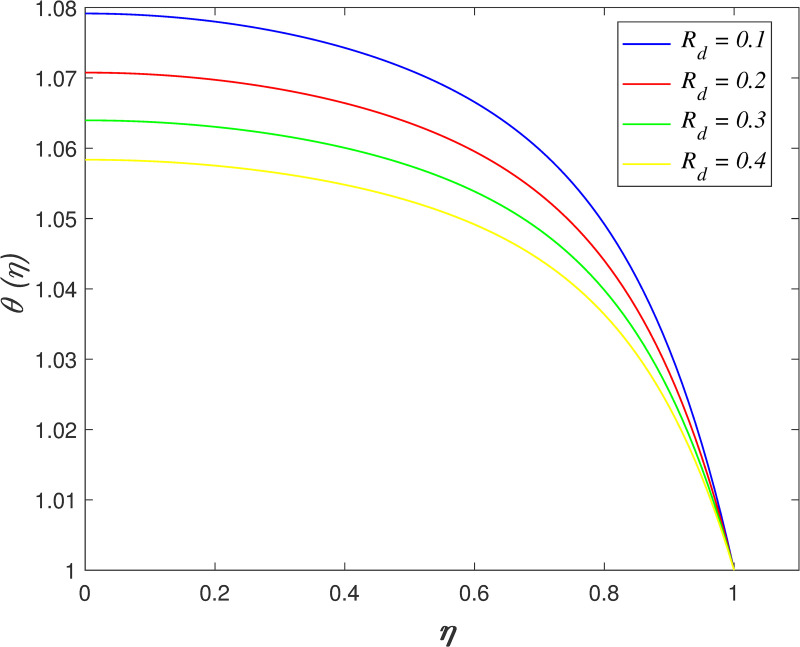
Impact of *R*_*d*_ on temperature.

**Fig 19:**
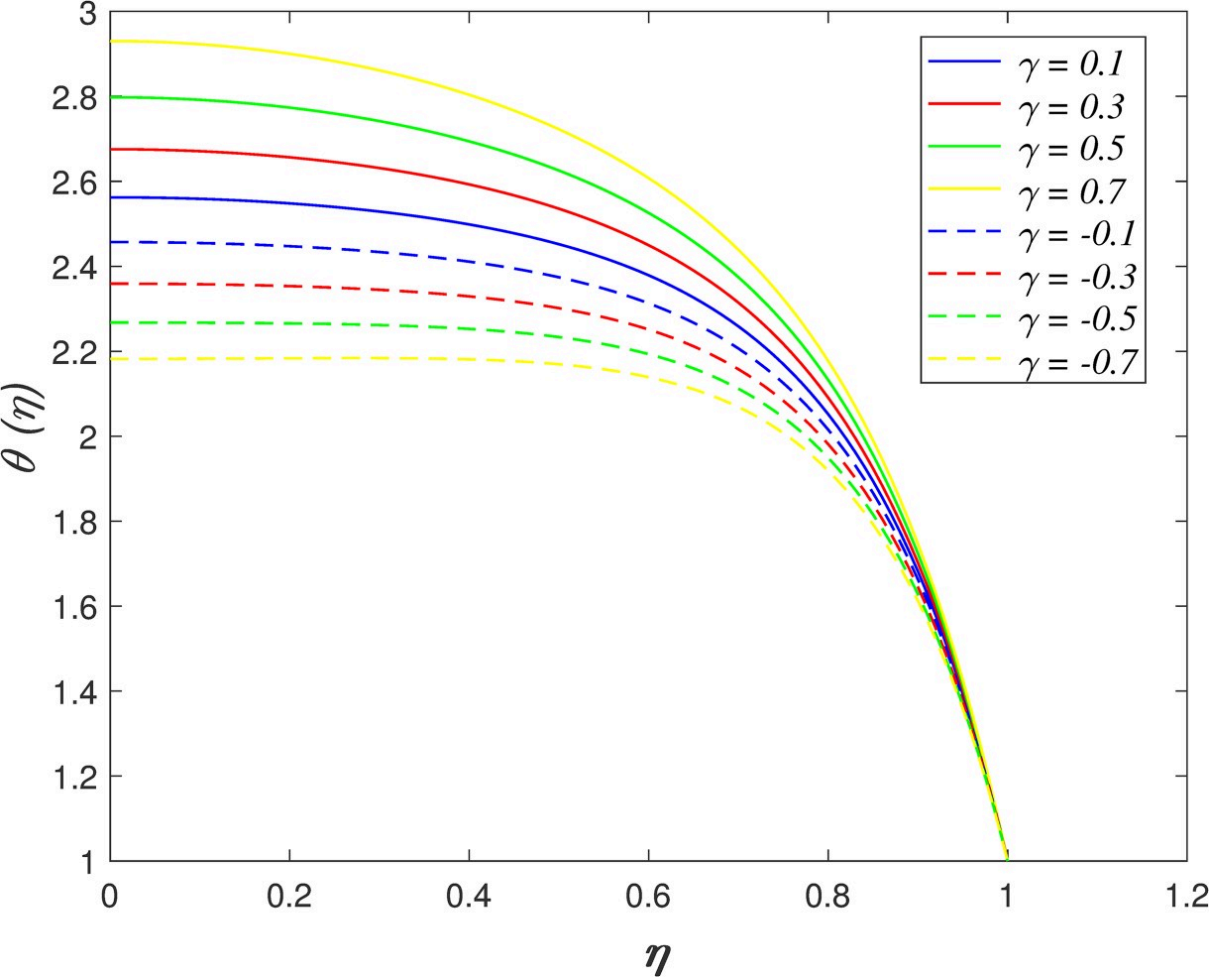
Impact of *γ* on temperature.

**Fig 20:**
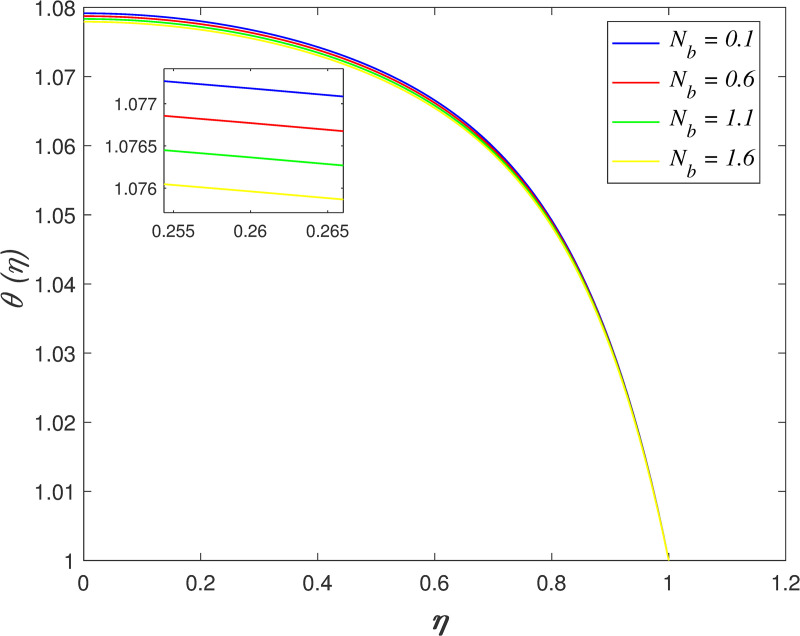
Impact of *N*_*b*_ on temperature.

**Fig 21:**
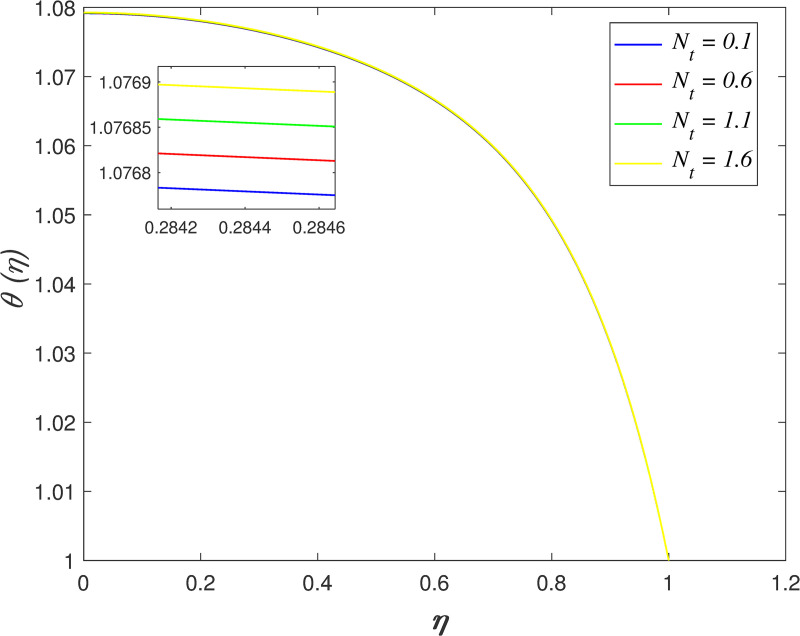
Impact of *N*_*t*_ on temperature.

Fig 16 discovers the variation of *Pr* on temperature field. The temperature of the flow boosts with raise in *Pr*. Prandtl number is the ratio of momentum diffusivity and thermal diffusivity. The increment of *Pr* cause the heat capacity of fluid increases. It has enhanced the heat absorption in the flow and thus, promotes the temperature in the fluid vicinity. The effect of *Ec* on temperature field is explored in Fig 17. It is observed that the flow temperature elevates for increasing *Ec*. The heat generated due to the motion of fluid particles in the flow with high viscosity is known as viscous dissipation. It is denoted by Eckert number. The raise in *Ec* elevates the kinetic energy of fluid particles, and it cause boosting the temperature in the flow. The impact of *R*_*d*_ on temperature field. Fig 18 describes the flow temperature declines because the transfer of thermal energy from fluid region to the upper wall elevates with higher values of *R*_*d*_. The influence of *γ* on temperature field is illustrated in Fig 19. It is notable that indicates heat sink and indicates heat source. The fluid temperature drops in the heat absorption case (*γ*<0) and it rises in the heat generation case *γ*>0. Heat generation boosts the thermal energy in the fluid vicinity and therefore, elevating the temperature profile. On the contrary, an opposite behaviour is shown in the heat sink case. The effect of *N*_*b*_ on temperature field is portrayed in Fig 20. It is discovered that the flow temperature drops as *N*_*b*_ rises. Brownian motion is a significant factor that enhances the thermal conductivity of nanofluid. It promotes the heat transfer from flow vicinity to the upper wall, which resulting in the temperature of the flow decreases. The variation of *N*_*t*_ on temperature field is demonstrated in Fig 21. The flow temperature elevates with enhancement of *N*_*t*_. The thermophoretic force in the nanofluid is induced by the temperature gradient within the flow and the upper wall. This force accelerates the kinetic energy of nanoparticles and consequently, increasing the temperature profile in the fluid vicinity.

**Fig 22:**
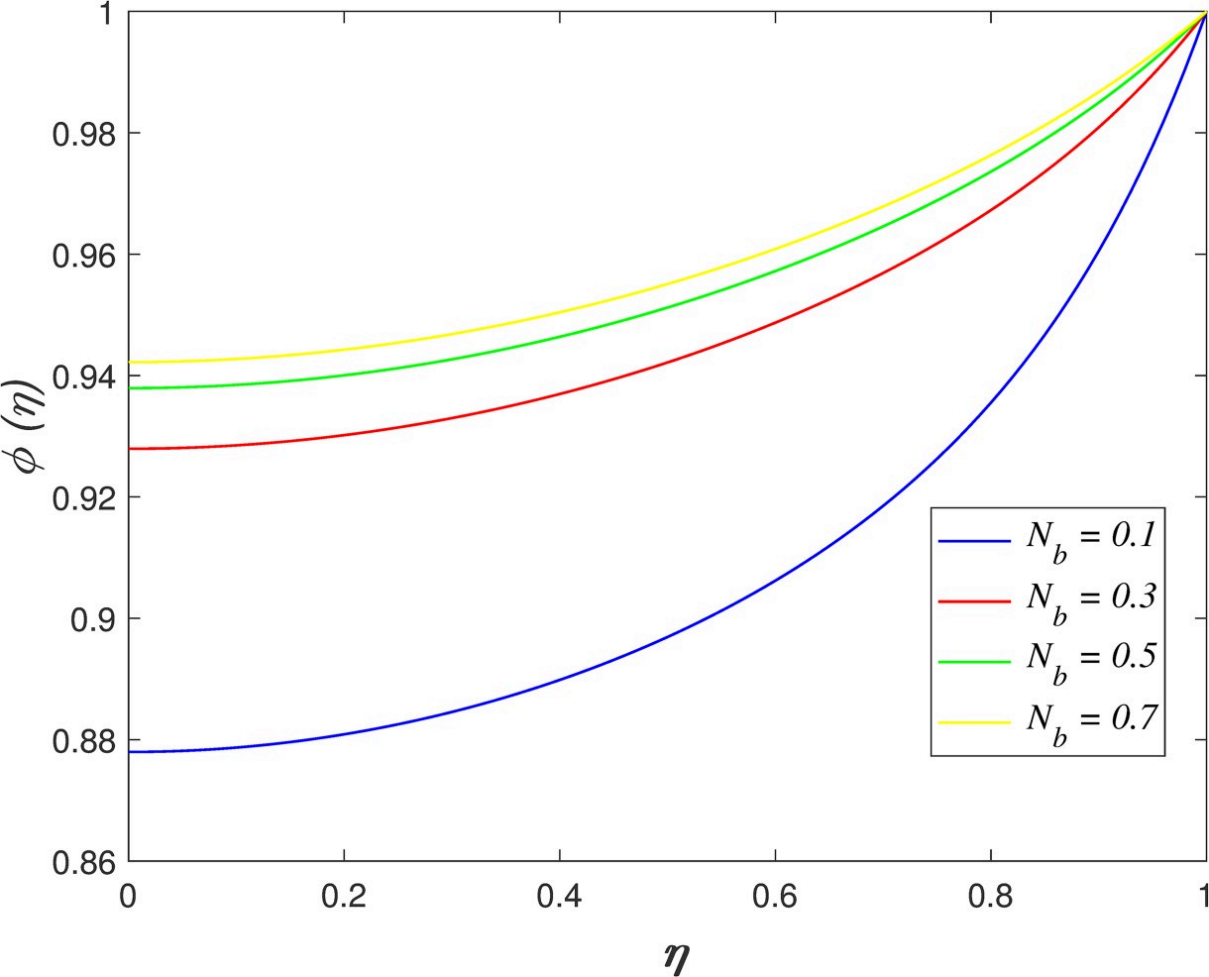
Impact of *N*_*b*_ on concentration.

**Fig 23:**
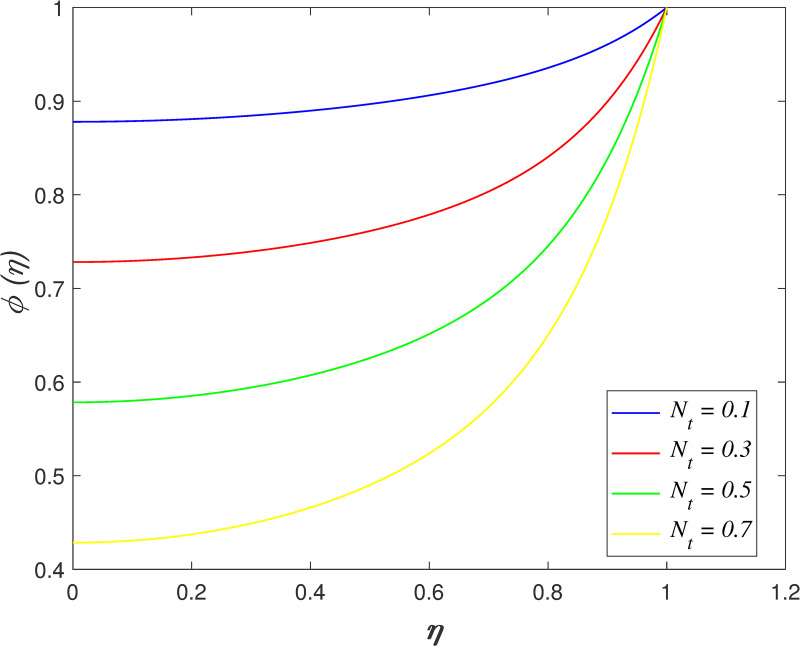
Impact of *N*_*t*_ on concentration.

**Fig 24:**
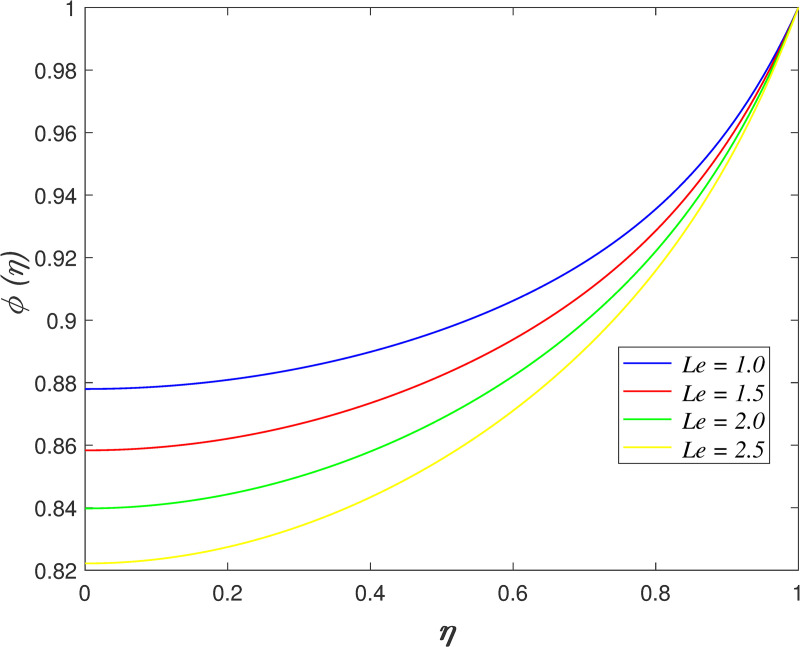
Impact of *Le* on concentration.

**Fig 25:**
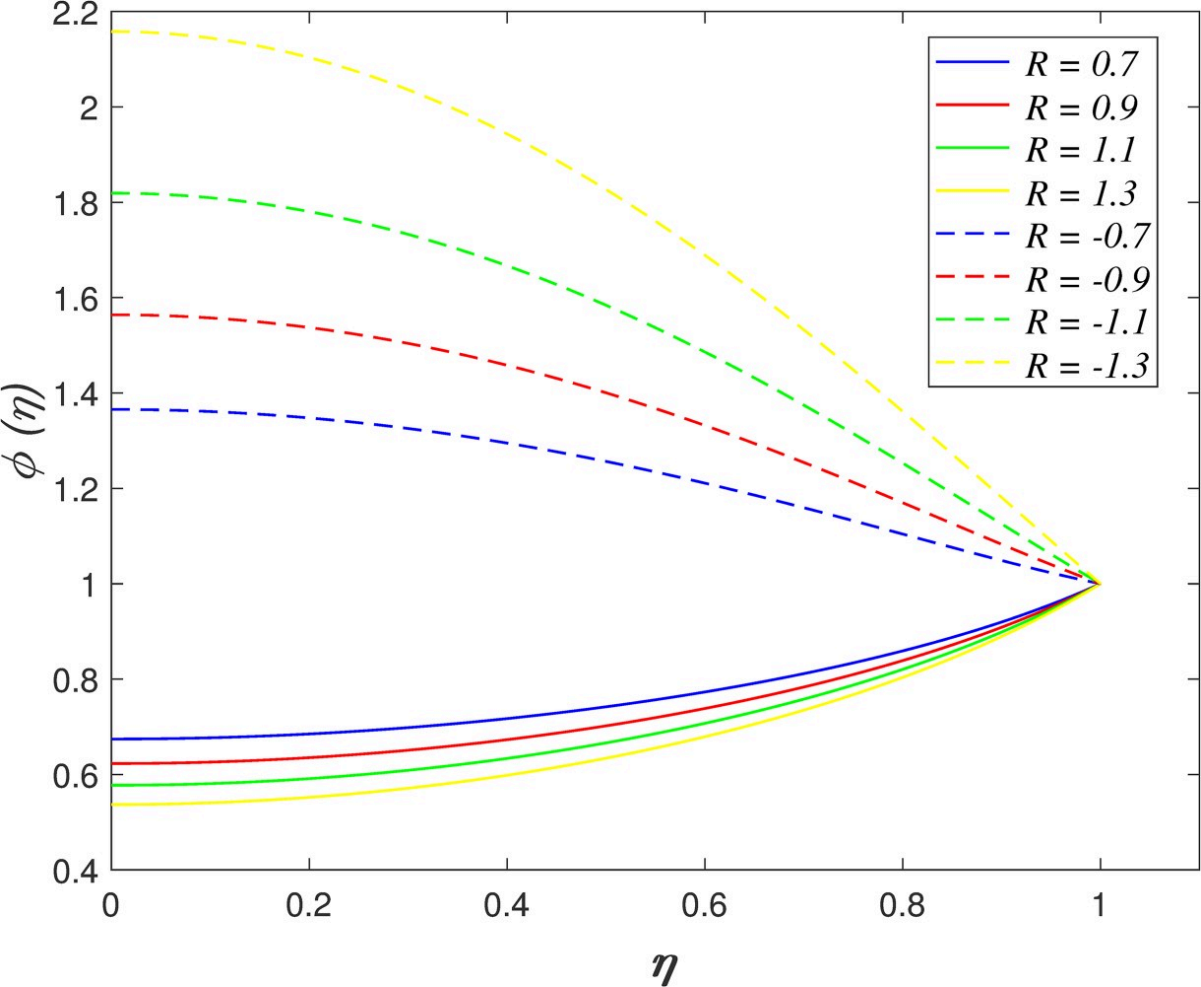
Impact of *R* on concentration.

Fig 22 portrays the effect of *N*_*b*_ on fluid concentration. The nanoparticles concentration increases as *N*_*b*_ rises. Physically, the kinetic energy of nanoparticles accelerates due to Brownian motion. The mass transfer from the upper boundary to the flow area increase the concentration field. The variation of *N*_*t*_ on concentration field is depicted in Fig 23. The reduction of nanoparticles concentration occurs with raise in *N*_*t*_. The thermophoretic force cause the mass transfer from the flow region to the upper boundary enhances. Fig 24 depicts the variation of *Le* on concentration field. It is noted that the nanoparticles concentration drops as *Le* rises. Lewis number is the ratio of thermal diffusion and mass diffusion. The mass diffusivity in the nanofluid decelerates with increment of *Le*, which resulting in decreases the concentration profile. Fig 25 illustrates the influence of *R* on concentration field. It is worth mentioning that the constructive and destructive chemical reaction are represented by *R*<0 and *R*>0, respectively. The concentration profile elevates when *R*<0 and it reduces when *R*>0. The constructive chemical reaction promotes the reaction rate in the nanofluid and thus, enhances the fluid concentration. Meanwhile, an opposite behaviour is found in the destructive chemical reaction case.

## 4. Physical quantities of interest

Skin friction coefficient, Nusselt and Sherwood numbers are the physical quantities of interest in the fluid flow. The friction of the fluid against the boundary plate is illustrated by skin friction parameter. Furthermore, Nusselt and Sherwood parameters are the non-dimensional parameter that represents the rate of thermal and mass transfer within the flow and boundary plate. The definition of skin friction coefficient *Cf*_*x*_, Nusselt number *Nu*_*x*_ and Sherwood number *Sh*_*x*_ are denoted by [64]

$$Cf_{x}=\frac{\tau_{w}}{\rho_{f}v_{w}^{2}},\;Nu_{x}=\frac{{lq}_{w}}{\alpha_{f}T_{w}},\;Sh_{x}=\frac{{lq}_{s}}{D_{B}C_{w}},$$

where *τ*_*w*_, *q*_*w*_ and *q*_*s*_ are the skin friction, heat and mass flux on the plate. The expressions of *τ*_*w*_, *q*_*w*_ and *q*_*s*_ are given as

$$\tau_{w}=\mu_{B}\left(1+\frac{1}{\lambda_{1}}\right){\left[\frac{\partial u}{\partial y}\right]}_{y=h(t)},\;q_{w}=-{\alpha_{f}(\frac{\partial T}{\partial y})}_{y=h(t)},\;q_{s}=-D_{B}{\left(\frac{\partial C}{\partial y}\right)}_{y=h(t)}.$$

The non-dimensional of *Cf*_*x*_, *Nu*_*x*_ and *Sh*_*x*_ are

$$\frac{l^{2}}{x^{2}}(1-\alpha t){Re}_{x}Cf_{x}=\left(1+\frac{1}{\lambda_{1}}\right)f^{\prime \prime }(1),$$

$$\sqrt{\left(1-\alpha t\right)}Nu_{x}=-\theta^{\prime }(1),$$

$$\sqrt{\left(1-\alpha t\right)}Sh_{x}=-\phi^{\prime }(1).$$

Numerical results of the physical quantities when varying the dimensionless parameters is depicted in Tables 4–7. Table 4 demonstrates the influence of *S* on skin friction coefficient, Nusselt and Sherwood numbers. It is found that the skin friction coefficient boosts as *S* rises, while contrary effect is noted in the Nusselt and Sherwood numbers. The internal friction of the fluid and the boundary surface elevates caused by the flow accelerates as the plates moving closer. Meanwhile, the deceleration of Nusselt and Sherwood numbers rate is caused by the kinetic energy of nanoparticles slows down in the narrow channel. The variation of *λ*_1_, *De*, *Da* and *Ha* on wall shear stress is presented in Table 5. The skin friction coefficient enhances for increasing *Ha*, while it drops with raise in *λ*_1_, *Da* and *De*. The velocity profile adjacent the upper boundary raises due to the Lorentz force, which result in enhancing the frictional force in the nanofluid. In contrast, the viscosity of Jeffrey fluid rises as *λ*_1_ and *De* increases, and thus the velocity decreases in the flow region. Moreover, the increment of *Da* reduce the flow adjacent the upper boundary. Consequently, the velocity profile declines lead to the drop of skin friction coefficient. Table 6 reveals the impact of *Ec*, *R*_*d*_, *γ*, *N*_*b*_ and *N*_*t*_ on rate of mass transfer. The ratio of heat transfer by convection and heat transfer by diffusion is represented by Nusselt number. It is noticed that the Nusselt number boost when *Ec*, *R*_*d*_, *γ* and *N*_*t*_ increases, while *N*_*b*_ decreases the rate of heat transfer. The raise in *Ec*, *R*_*d*_, *γ* and *N*_*t*_ elevate the fluid temperature, which cause the kinetic energy of nanoparticles enhancing in the boundary region. This phenomenon increases the Nusselt number. On the contrary, it is noticed on Fig 20 that the fluid temperature drops as Brownian motion rises. The kinetic energy in the flow declines and thus, reducing the convective heat transfer. The effect of *Le*, *R*, *N*_*b*_ and *N*_*t*_ on rate of mass transfer is displayed in Table 7. Sherwood number enhances when *Le*, *R* and *N*_*t*_ rises, whereas it slows down for increasing *N*_*b*_ values. Sherwood number is the ratio of convective mass transfer and diffusive mass transfer. Physically, the acceleration of Brownian motion increases the diffusive mass transfer, which resulting in the nanoparticles concentration in the flow enhances as exhibited in Fig 21. Sherwood number is inversely proportional to the diffusive mass transfer. Consequently, this behaviour has caused the Sherwood number declines. Meanwhile, the opposite behaviour is observed for increasing of *Le*, *R* and *N*_*t*_. The increment of Sherwood number indicates that *Le*, *R* and *N*_*t*_ boosts the mass transfer caused by convection.

**Table 4:** Numerical results of –(1+1/*λ*_1_)*f*′′(1), −*θ*′(1) and *ϕ*′(1) for *S* as *γ* = *De* = 0.01, *Ec* = *Ha* = *R*_*d*_ = *N*_*b*_ = *N*_*t*_ = 0.1, *λ*_1_ = *Da* = *δ* = 1 and *Le* = *R* = *Pr* = 1.5.

*S*	–(1+1/*λ*_1_)*f*′′(1)	−(1+4/3 *R*_*d*_)*θ*′(1)	*ϕ*′(1)
-2.5	4.430668	3.163448	3.506953
-2.0	4.886665	2.905970	3.238322
-1.5	5.305616	2.704779	3.023363
-1.0	5.693824	2.544538	2.847973
-0.5	6.056136	2.414910	2.702511
0	6.396352	2.308649	2.580162
0.5	6.717502	2.220555	2.475995
1.0	7.022035	2.146812	2.386363
1.5	7.311961	2.084569	2.308519
2.0	7.588944	2.031656	2.240360
2.5	7.854379	1.986400	2.180247

**Table 5:** Numerical outputs of –(1+1/*λ*_1_)*f*′′(1) for *λ*_1_, *Ha*, *Da* and *De* when *γ* = 0.01, *Ec* = *R*_*d*_ = *N*_*b*_ = *N*_*t*_ = 0.1, *S* = *δ* = 1 and *Le* = *R* = *Pr* = 1.5.

***λ***_**1**_	***Ha***	***Da***	***De***	**–(1+1/*λ***_**1**_**)*f*′′(1)**
1.0	0.1	1	0.01	7.022035
1.5				5.949993
2.0				5.413036
2.5				5.090449
3.0				4.875182
3.5				4.721301
1.5	1.0	1.0	0.01	6.112595
	1.5			6.312200
	2.0			6.581781
	2.5			6.913097
	3.0			7.297395
	3.5			7.726136
1.5	0.1	1.0	0.01	5.949993
		1.5		5.856893
		2.0		5.809823
		2.5		5.781412
		3.0		5.762399
		3.5		5.748783
1.5	0.1	1.0	0.010	5.949993
			0.011	5.949495
			0.012	5.948999
			0.013	5.948502
			0.014	5.948006

**Table 6:** Numerical outputs of $-(1+\frac{4}{3}R_{d})$ *θ*′(1) for *Ec*, *R*_*d*_, *γ*, *N*_*b*_ and *N*_*t*_ when *De* = 0.01, *Ha* = 0.1, *S* = *Da* = *δ* = 1 and *λ*_1_ = *Pr* = *Le* = *R* = 1.5.

*Ec*	*R*_*d*_	*γ*	*N*_*b*_	*N*_*t*_	$-(1+\frac{4}{3}R_{d})\theta^{\prime }(1)$
0.1	0.1	0.01	0.1	0.1	1.781145
0.2					3.667674
0.3					5.688882
0.4					7.864110
0.5					10.217443
0.6					12.778246
0.1	0.1	0.01	0.1	0.1	1.781145
	0.2				1.792616
	0.3				1.802846
	0.4				1.811952
	0.5				1.820069
	0.6				1.827329
0.1	0.1	-0.9	0.1	0.1	0.409998
		-0.6			0.768647
		-0.3			1.205566
		0.3			2.505041
		0.6			3.599851
		0.9			5.494037
0.1	0.1	0.01	0.2		1.697410
			0.4		1.544461
			0.6		1.408850
			0.8		1.288430
			1.0		1.181336
0.1	0.1	0.01	0.1	0.2	1.841413
				0.4	1.979279
				0.6	2.147203
				0.8	2.357970
				1.0	2.633940

**Table 7:** Numerical outputs of *ϕ*′(1) for *Le*, *R*, *N*_*b*_ and *N*_*t*_ when *γ* = *De* = 0.01, δ = *S* = *Da* = 1, *Ha* = *Ec* = *R*_*d*_ = 0.1 and *Pr* = *λ*_1_ = 1.5.

*Le*	*R*	*N*_*b*_	*N*_*t*_	*ϕ*′(1)
0.5	1.5	0.1	0.1	1.846706
1.0				2.040151
1.5				2.202992
2.0				2.346816
2.5				2.477629
3.0				2.598894
1.5	-1.5	0.1	0.1	-2.087331
	-1.0			0.288282
	-0.5			1.044010
	0.5			1.766023
	1.0			2.002534
	1.5			2.202992
1.5	1.5	0.2	0.1	1.719392
		0.4		1.480209
		0.6		1.402551
		0.8		1.365073
		1.0		1.343527
1.5	1.5	0.1	0.2	3.191918
			0.4	5.446726
			0.6	8.177951
			0.8	11.579770
			1.0	15.985429

## 5. Conclusions

The impacts of chemical reaction and thermal radiation on unsteady hydromagnetic squeezing flow of Jeffrey nanofluid in a porous channel with heat source/sink was analysed. The presence of joule heating and viscous dissipation is considered. The governing equations is solved using Keller-box scheme and then, the numerical and graphical results are attained via MATLAB software. Comparison of the present results with previous published results is carried out. It is discovered in excellent agreement. Physically, the influences of *S*, *λ*_1_, *Ha*, *Da*, *De*, *δ*, *Pr*, *Ec*, *R*_*d*_, *γ*, *Le*, *N*_*b*_, *N*_*t*_ and *R* on velocity, temperature and nanoparticles concentration are examined. The significant results from the analysis are summarized as:

1. The flow velocity increases as the surfaces moving closer (*S*>0) and it decreases as the plates moving apart (*S*<0) nearer the upper plate vicinity.
2. The wall shear stress accelerates for increasing *Ha* and *S*, whereas it drops with raise in *λ*_1_, *De* and *Da*.
3. The reduction of flow velocity, temperature, and concentration is noticed as *λ*_1_ and *Ha* increases.
4. The velocity profile adjacent the upper plate vicinity decelerates for increasing *Da* and *De*.
5. The temperature profile and heat transfer rate boost with raise in *Pr*, *Ec*, *γ* and *N*_*t*_, whereas opposite impact is observed when *N*_*b*_ increases.
6. The increment of *R*_*d*_ reducing the temperature profile and it enhancing the heat transfer rate.
7. The nanoparticles concentration drops, and the mass transfer rate accelerates for increasing *R*, *Le* and *N*_*t*_.
8. The increase in *N*_*b*_ enhance the nanoparticles concentration and decline the mass transfer rate.
